# Supplementary material for: PRPF8-mediated dysregulation of hBrr2 helicase disrupts human spliceosome kinetics and 5´-splice-site selection causing tissue-specific defects
Source: Nat Commun. 2024 Apr 11;15:3138. doi: 10.1038/s41467-024-47253-0 (PMC11009313; doi:10.1038/s41467-024-47253-0)
Supplement: Supplementary file 1 — Supplementary Information [file 41467_2024_47253_MOESM1_ESM.pdf]

Supplementary Material for

PRPF8-mediated dysregulation of hBrr2 helicase disrupts human spliceosome kinetics and 5´-splice-site selection causing tissue-specific defects

Robert Atkinson[1*], Maria Georgiou[1*], Chunbo Yang[1*], Katarzyna Szymanska[2*], Albert Lahat[3*], Elton J. R. Vasconcelos[4*], Yanlong Ji[5,6], Marina Moya Molina[1,7], Joseph Collin[1], Rachel Queen[1], Birthe Dorgau[1], Avril Watson[1,7], Marzena Kurzawa-Akanbi[1], Ross Laws[8], Abhijit Saxena[1], Chia Shyan Beh[1], Chileleko Siachisumo[1], Franziska Goertler[9], Magda Karwatka[2], Tracey Davey[8], Chris F Inglehearn[2], Martin McKibbin[2], Reinhard Lührmann[5], David H Steel[1], David J Elliott[1], Lyle Armstrong[1], Henning Urlaub[5,6,10], Robin R Ali[11#], Sushma-Nagaraja Grellscheid[3,9#], Colin A Johnson[2#], Sina Mozaffari-Jovin[5,12#], Majlinda Lako[1#]

1 Biosciences Institute, Newcastle University, UK
2 Leeds Institute of Medical Research, University of Leeds, UK
3 Department of Biosciences, Durham University, UK
4 Leeds Omics, University of Leeds, UK
5 Max-Planck-Institute for Multidisciplinary Sciences, Göttingen, Germany
6 Institute of Clinical Chemistry, University Medical Center Göttingen, Germany
7 Newcells Biotech, UK
8 Electron Microscopy Research Services, Newcastle University, UK
9 Department of Informatics, University of Bergen, Norway
10 Göttingen Center for Molecular Biosciences, Georg August University of Göttingen, Germany
11 Centre for Gene Therapy and Regenerative Medicine, Kings College London, UK
12 Department of Medical Genetics and Medical Genetics Research Center, School of Medicine, Mashhad University of Medical Sciences, Mashhad, Iran

* These authors contributed equally
# These authors jointly supervised this work

This PDF file includes:
Supplementary Figures S1 to S10
Supplementary data legends
Supplementary Methods
Error bar information for Figure 2F
Supplementary Table 1
Supplementary References

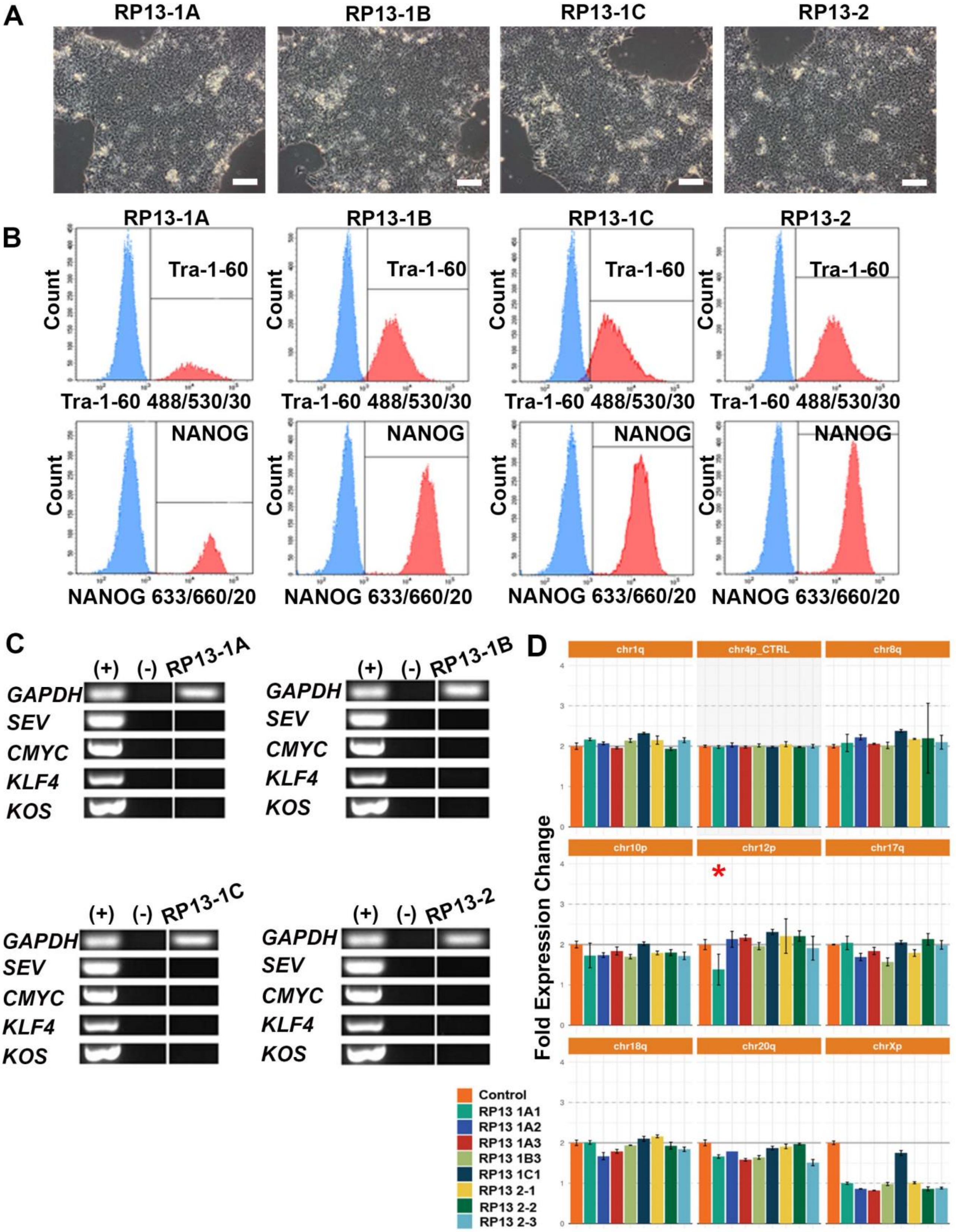
A
RP13-1A
RP13-1B
RP13-1C
RP13-2
B
Tra-1-60
Count
Tra-1-60 488/530/30
NANOG
NANOG 633/660/20
C
(+)
(-)
GAPDH
SEV
CMYC
KLF4
KOS
D
chr1q
chr4p_CTRL
chr8q
chr10p
chr12p
chr17q
chr18q
chr20q
chrXp
Fold Expression Change
Control
RP13 1A1
RP13 1A2
RP13 1A3
RP13 1B3
RP13 1C1
RP13 2-1
RP13 2-2
RP13 2-3

Figure S1

Figure S1: Generation and characterisation of RP13-iPSCs. A) Representative microphotographs showing typical iPSC morphology in RP13-iPSCs, scale bars 100 µm; **B**) Flow cytometric analysis of TRA-1-60 and NANOG. All the iPSC lines show single peaks of positive populations accounting for more than 95% of all the cells examined. Representative images of 3 replicates are shown with the red shaded area indicating the antibody immunostained cells and the blue shaded area the isotype control-stained cells; **C**) Verification of Sendai virus genome (*SEV*) and the transgenes (*KOS, KLF4, C-MYC*) clearance by RT-PCR; **D**) Genome stability analysis of iPSCs carried out with the hPSC Genetic Analysis kit showing comparable copy number with control in all observed loci except for RP13-1A1, which showed a decreased copy number in the Chr12p region as marked by *.

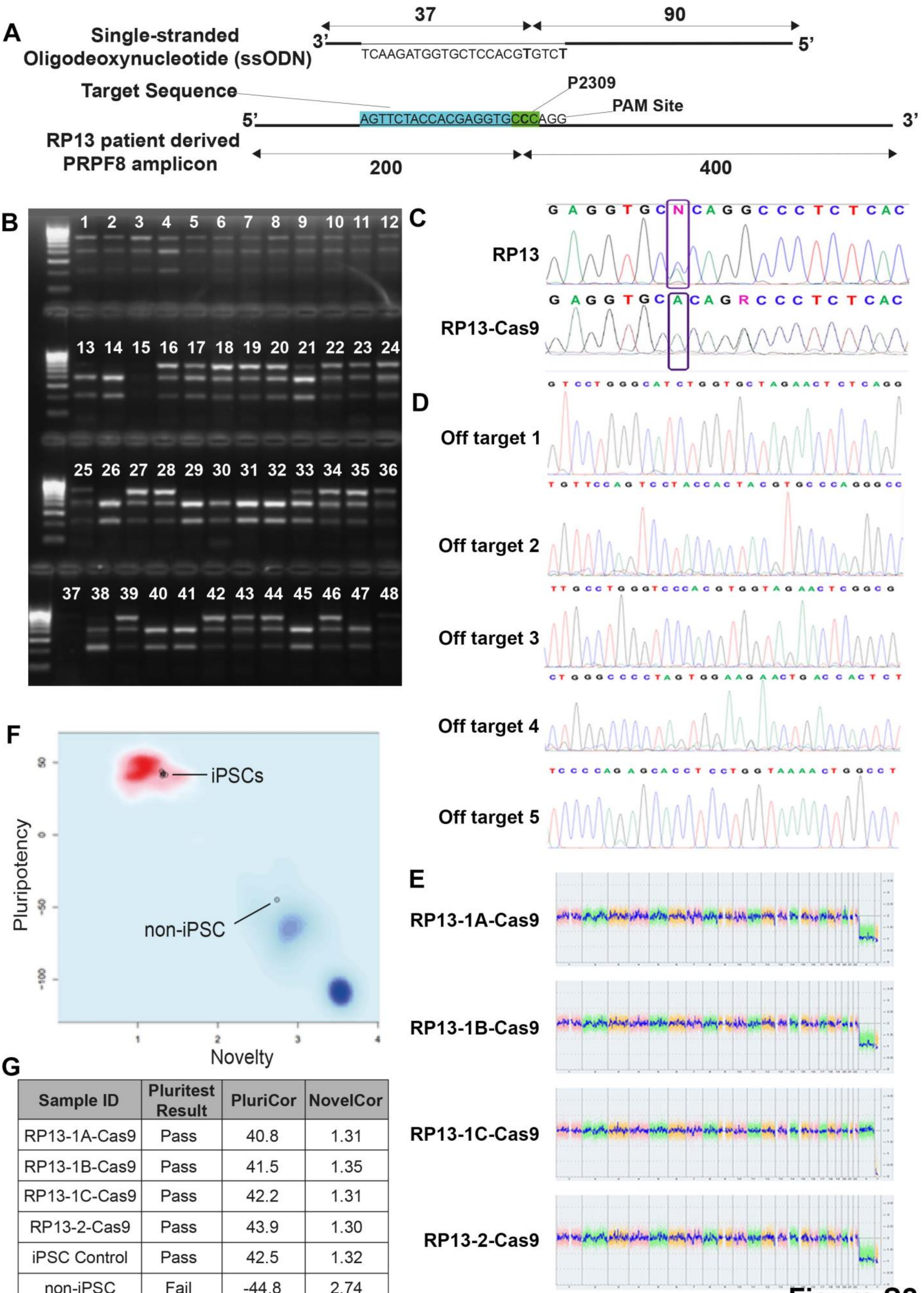

Sample ID	Pluritest Result	PluriCor	NovelCor
RP13-1A-Cas9	Pass	40.8	1.31
RP13-1B-Cas9	Pass	41.5	1.35
RP13-1C-Cas9	Pass	42.2	1.31
RP13-2-Cas9	Pass	43.9	1.30
iPSC Control	Pass	42.5	1.32
non-iPSC	Fail	-44.8	2.74

Figure S2

Figure S2: *PRPF8* mutation correction by CRISPR/Cas9 gene editing. A) Diagram showing the design of asymmetric ssODN aligned to the PRPF8 genomic sequence and guide RNA (gRNA; cyan); **B**) *Apa*L1 restriction enzyme analysis showing the digest of 602 bp fragment into 206 and 396 bp band in the successfully corrected clones; **C**) Representative DNA sequence of RP13-iPSC and isogenic CRISPR/Cas9 control showing the correction of heterozygous mutation to the homozygous wild type nucleotide: N indicates where a nucleotide was not automatically assigned, and R indicates the base could be either C or A; **D**) Representative DNA sequences of all five tested off-target sites showing no deviations from the wild type sequence; **E**) Karyostat genome stability assay excluded chromosomal aberrations in CRISPR/Cas9 corrected iPSC lines. The whole genome view displays all somatic and sex chromosomes in one frame with high level copy number. A value of 2 represents a normal copy number state (CN = 2), value of 3 represents chromosomal gain (CN = 3), value of 1 represents a chromosomal loss (CN = 1). The pink, green and yellow colours indicate the raw signal for each individual chromosome probe, while the blue represents the normalized probe signal used to identify copy number and aberrations (if any); **F, G**) Table and accompanying plot of results from PluriTest assay. The red and blue areas of the plot show how the tested samples compare with reference pluripotent and non-pluripotent cell types, respectively. PluriCor and NovCor were determined using an algorithm that integrates gene expression data to authenticate pluripotency status. In brief, PluriCor is a score of pluripotency whereas NovCor increases when non-pluripotent cell types are also present. A non-iPSC sample was used in this experiment to serve as a negative control for non-pluripotency.

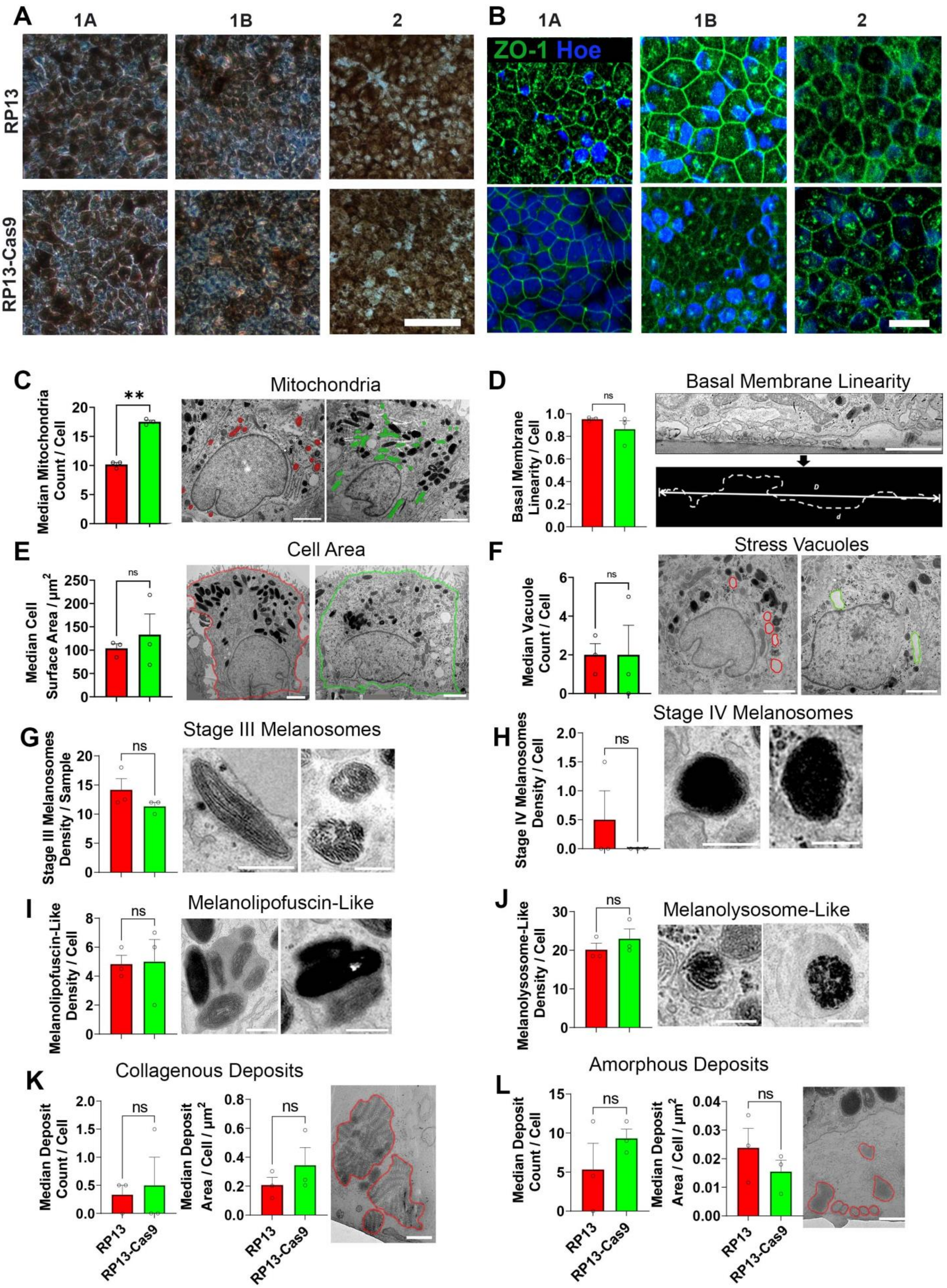

A
1A
1B
2
RP13
RP13-Cas9
B
ZO-1 Hoe
C
Mitochondria
Median Mitochondria Count / Cell
**
D
Basal Membrane Linearity
Basal Membrane Linearity / Cell
ns
E
Cell Area
Median Cell Surface Area / µm²
F
Stress Vacuoles
Median Vacuole Count / Cell
G
Stage III Melanosomes
Stage III Melanosomes Density / Sample
H
Stage IV Melanosomes
Stage IV Melanosomes Density / Cell
I
Melanolipofuscin-Like
Melanolipofuscin-Like Density / Cell
J
Melanolysosome-Like
Melanolysosome-Like Density / Cell
K
Collagenous Deposits
Median Deposit Count / Cell
Median Deposit Area / Cell / µm²
L
Amorphous Deposits
Median Deposit Count / Cell
Median Deposit Area / Cell / µm²

Figure S3: Structural and ultrastructural characterisation of iPSC-RPE cells. **A**) Brightfield microscopy of iPSC-RPE monolayers showing typical cobblestone appearance. Scale bar 50 µm; **B**) ZO-1 staining of tight junctions showing hexagonal RPE cell morphology. Scale bar 20 µm; **C**) Bar chart of mitochondria number per cell showing significant (p<0.01) decrease in RP13 (10.4±0.3) compared to RP13-Cas9 iPSC-RPE cells (16.9±0.7); **D**) Bar chart showing unaffected basal membrane linearity of RP13 (0.95±0.01) and RP13-Cas9 (0.86±0.07) iPSC-RPE. TEM image shows infoldings of basal cell membranes. Cell width was measured as a linear distance (D), membrane by freehand trace (d), where linearity=(d/D); **E**) The mean surface area of RP13 (101± 7µm^2) and RP13-Cas9 (127±32 µm^2) iPSC-RPE; **F**) Bar chart showing unaffected median vacuole counts per cell for RP13 (2.1±0.4) and RP13-Cas9 iPSC-RPE (2.5±1.2). TEM images highlight relevant features in RP13 (red) and RP13-Cas9 (green) in (A, B, D); **G**) Bar chart showing unaffected density of stage III melanosomes per cell in RP13 (14.2±1.9) and RP13-Cas9 iPSC-RPE (11.3±0.67); **H**) Bar chart showing unaffected density of stage IV melanosomes per cell in RP13 (0.5±0.5) and RP13-Cas9 iPSC-RPE (0±0); **I**) Bar chart showing unaffected density of melanolipofuscin-like complexes per cell in RP13 (4.8±0.6) and RP13-Cas9 iPSC-RPE (5.0±1.5); **J**) Bar chart showing unaffected density of melanolysosome-like complexes per cell in RP13 (20.2±1.7) and RP13-Cas9 iPSC-RPE (23.0±2.5). Representative images are shown on right-hand side of (G, H, I, J); **K**) Bar charts showing no significant difference in the number and area of collagenous deposits per cell in RP13 and RP13-Cas9 iPSC-RPE. TEM image of collagenous deposits (red outline) with characteristic banded appearance; **L**) Bar charts showing no significant difference in the number and area of amorphous deposits per cell in RP13 and RP13-Cas9 iPSC-RPE. TEM image of an amorphous deposit (red outline), showing rounded electron dense appearance. Scale bars: 2 µm (A, B, C, D), 0.5 µm (D, E, F, G) and 1 µm (I, J). C-L, n = 3, where n is the median value per donor calculated from 10 cells per group. Results are presented as mean ± SEM. Statistical significance was assessed using a 2-tailed paired t-test.

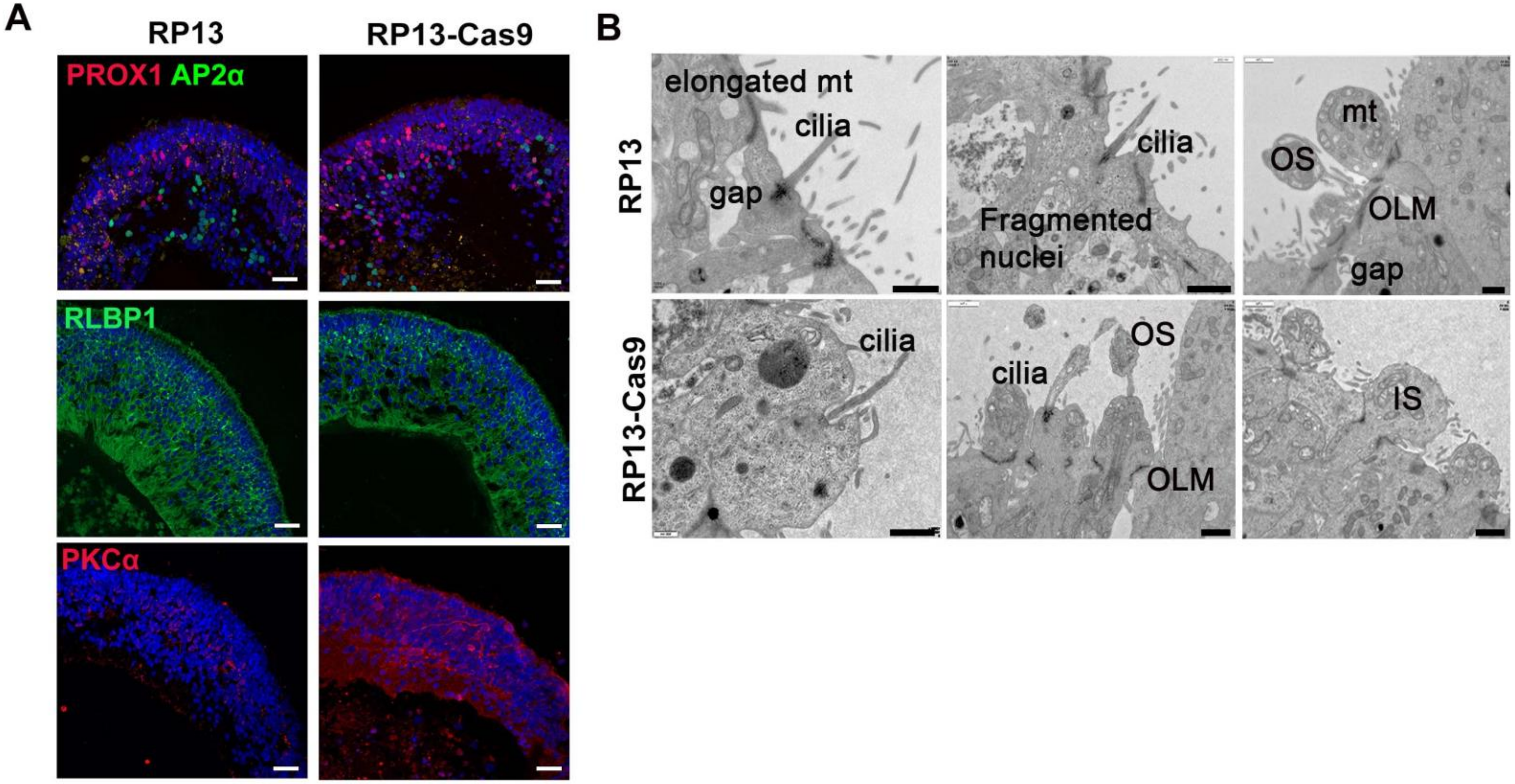
A
RP13
RP13-Cas9
PROX1 AP2α
RLBP1
PKCα
B
RP13
RP13-Cas9
elongated mt
cilia
gap
cilia
Fragmented
nuclei
mt
OS
OLM
gap
cilia
cilia
OS
OLM
IS

Figure S4

Figure S4. Additional characterisation of RP13-ROs. A) Immunostaining for horizontal (PROX1), amacrine (AP2α), Müller glia (RLBP1), and bipolar (PKCα) cell markers (scale bar = 50 μm). Both RP13 and RP13-Cas9 ROs contained horizontal, amacrine and bipolar cells in the putative inner nuclear layer; **B**) Representative TEM images indicated similar ultrastructural morphologies in RP13 and RP13-Cas9 ROs (including typical rounded inner segments, connecting cilia, photoreceptor-like outer segments and an outer limiting membrane), but showed the presence of intracellular gaps and fragmented nuclei in RP13 photoreceptors (scale bars = 1 μm).

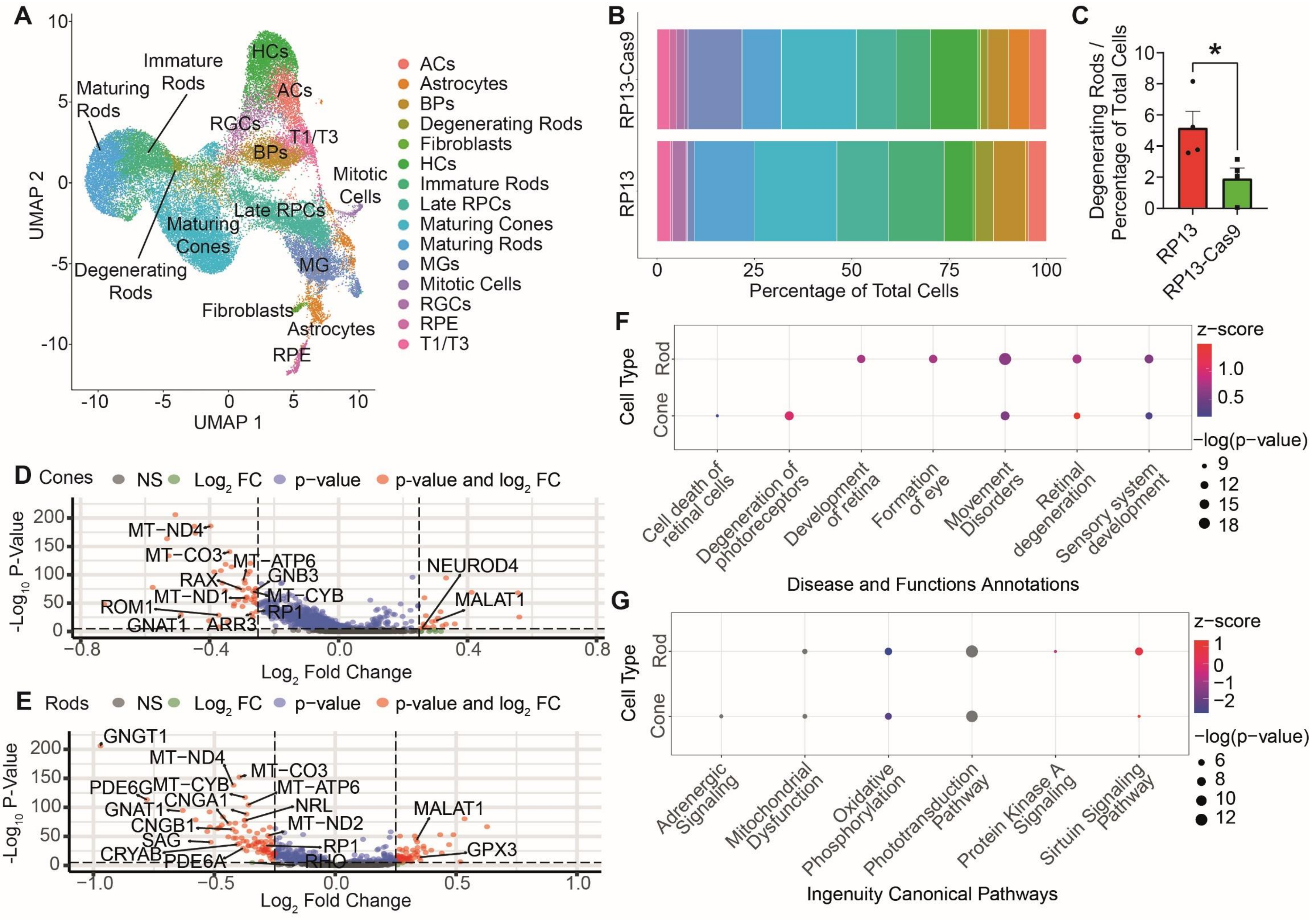
A
B
C
D
E
F
G
Maturing Rods
Immature Rods
HCs
ACs
RGCs
T1/T3
BPs
Mitotic Cells
Late RPCs
Maturing Cones
Degenerating Rods
MG
Fibroblasts
Astrocytes
RPE
UMAP 1
UMAP 2
ACs
Astrocytes
BPs
Degenerating Rods
Fibroblasts
HCs
Immature Rods
Late RPCs
Maturing Cones
Maturing Rods
MGs
Mitotic Cells
RGCs
RPE
T1/T3
RP13-Cas9
RP13
Percentage of Total Cells
Degenerating Rods / Percentage of Total Cells
*
RP13
RP13-Cas9
Cones
NS
Log2 FC
p−value
p−value and log2 FC
-Log10 P-Value
Log2 Fold Change
MT−ND4
MT−CO3
MT−ATP6
RAX
GNB3
MT−ND1
MT−CYB
ROM1
RP1
GNAT1
ARR3
NEUROD4
MALAT1
Rods
GNGT1
MT−ND4
MT−CO3
PDE6G
MT−CYB
MT−ATP6
CNGA1
NRL
GNAT1
MT−ND2
CNGB1
SAG
RP1
CRYAB
PDE6A
RHO
MALAT1
GPX3
Cell Type
Rod
Cone
z−score
−log(p−value)
Cell death of retinal cells
Degeneration of photoreceptors
Development of retina
Formation of eye
Movement Disorders
Retinal degeneration
Sensory system development
Disease and Functions Annotations
Adrenergic Signaling
Mitochondrial Dysfunction
Oxidative Phosphorylation
Phototransduction Pathway
Protein Kinase A Signaling
Sirtuin Signaling Pathway
Ingenuity Canonical Pathways

Figure S5. Single cell (sc) RNA-Seq of day 210 RP13 and control ROs. A) UMAP visualisation of scRNA-Seq data showing the presence of all retina cell types (late retinal progenitor cells, transient neurogenic progenitors, mitotic cells, rods, cone, amacrine, retinal ganglion, horizontal, bipolar and Muller glia cells), alongside small fractions of RPE cells, astrocytes and fibroblasts; **B**) Side-by-side comparison of cell type percentage in control and RP13 organoids; **C**) A higher fraction of degenerating rods is present in RP13 organoids, data shown as mean ± SEM, * $p < 0.05$, pair-wise Student t-test; **D, E**) Volcano plots showing differentially expressed genes between RP13 cones (D) and rods (E) and isogenic control cells. Grey points represent genes that do not exceed the $-\log_{10}$ p-value or $\log_2$ fold change thresholds (1.3 and 0.25, respectively). Green points meet $\log_2$ fold change threshold, blue points meet $-\log_{10}$ p-value threshold, and red points meet both $\log_2$ fold change and $\log_{10}$ p-value thresholds. Key components of the mitochondrial respiratory chain complex I (*MT-ND1, MT-ND4*) and complex IV (*MT-CO3*), and electron transport-coupled proton transport (*MT-CO1, MT-CYB*) were significantly down-regulated in both RP13 cone and rod photoreceptors. This could reflect reduced mitochondria incidence in RP13 photoreceptors, or a more general mitochondrial dysfunction shown in panel G. **F, G)** Enriched disease processes and canonical pathways respectively in RP13 cone and rod photoreceptors revealed by the Ingenuity Pathway Analysis (IPA) analysis. The dot colour indicates the z-score assigned by Ingenuity Pathway Analysis to each pathway, where no z-score could be assigned the dot is coloured grey.

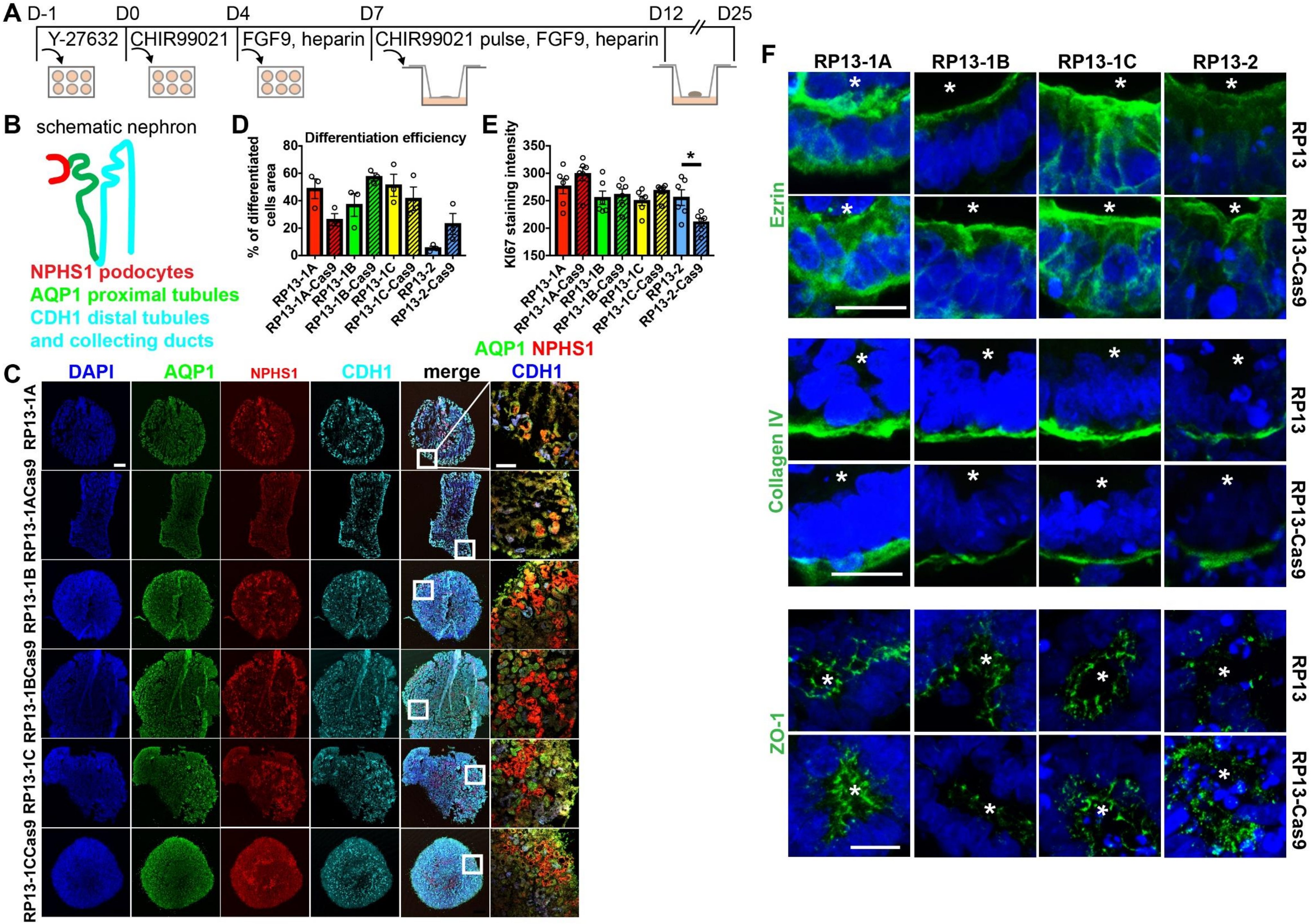
A
D-1
D0
D4
D7
D12
D25
Y-27632
CHIR99021
FGF9, heparin
CHIR99021 pulse, FGF9, heparin
B
schematic nephron
NPHS1 podocytes
AQP1 proximal tubules
CDH1 distal tubules
and collecting ducts
D
Differentiation efficiency
% of differentiated cells area
RP13-1A
RP13-1A-Cas9
RP13-1B
RP13-1B-Cas9
RP13-1C
RP13-1C-Cas9
RP13-2
RP13-2-Cas9
E
KI67 staining intensity
C
DAPI
AQP1
NPHS1
CDH1
merge
AQP1 NPHS1
CDH1
RP13-1A
RP13-1ACas9
RP13-1B
RP13-1BCas9
RP13-1C
RP13-1CCas9
F
RP13-1A
RP13-1B
RP13-1C
RP13-2
Ezrin
Collagen IV
ZO-1
RP13
RP13-Cas9

Figure S6. Generation and characterisation of RP13 KiOs. A) Schematic illustration of iPSCs differentiation into KiOs in six-well plates followed by culture in inserts on air-liquid interphase; **B)** Schematic illustration of kidney nephron with major markers including NPHS1 (podocytes; red), AQP1 (proximal tubules; green), CDH1 (distal tubules and collecting ducts; cyan); **C)** These markers were used to stain KiO sections. Scale bars are 500 µm for whole organoids and 20 µm for magnified insets indicated by white frames; **D)** Quantitative immunofluorescence analysis of differentiation of KiOs, indicating variable levels of efficiency. Data shown as mean ± SEM, n = 3; **E)** Quantitative immunofluorescence analysis of Ki67, a marker of proliferating cells. All lines other than RP13-2 KiOs display clear tubular structures, likely due to increased cell proliferation leading to compromised differentiation efficiency. Data shown as mean ± SEM, n = 5. Statistical significance was assessed using unpaired two-tailed t-test with Welch's correction, * indicates $p < 0.05$; **F)** Cell polarity markers in RP13 KiOs. RP13-Cas9 and RP13 KiOs were assessed for apical cell marker (ezrin, green), basal cell surface marker (collagen IV, green), and tight junction marker (ZO-1, green). Asterisks identify the KiO tubule lumen. There were no differences observed in these markers' localisation between control and patient organoids. Scale bars = 10 µm.

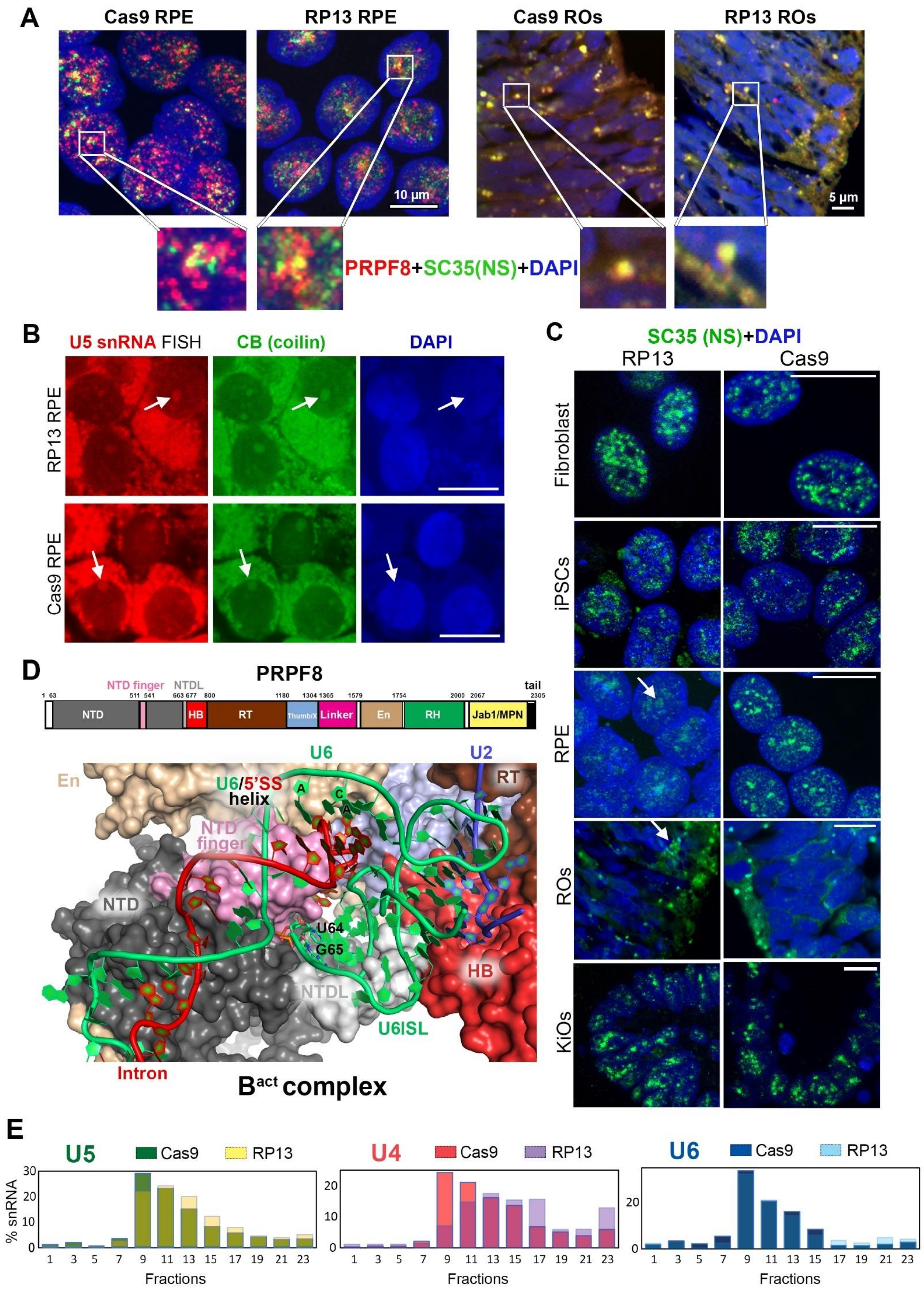
A
Cas9 RPE
RP13 RPE
Cas9 ROs
RP13 ROs
10 µm
5 µm
PRPF8+SC35(NS)+DAPI
B
U5 snRNA FISH
CB (coilin)
DAPI
RP13 RPE
Cas9 RPE
C
SC35 (NS)+DAPI
RP13
Cas9
Fibroblast
iPSCs
RPE
ROs
KiOs
D
PRPF8
NTD finger
NTDL
tail
1 63 511 541 663 677 800 1180 1304 1365 1579 1754 2000 2067 2305
NTD
HB
RT
Thumb/X
Linker
En
RH
Jab1/MPN
U6
U2
En
RT
U6/5'SS helix
NTD finger
NTD
U64
G65
NTDL
HB
U6ISL
Intron
B^act complex
E
U5
Cas9
RP13
U4
U6
% snRNA
Fractions

Figure S7: Analysis of PRPF8 localisation to splicing speckles. A) Co-staining of PRPF8 (red) and splicing speckles (SC35, green) followed by counterstaining by DAPI in RPE and ROs showing localisation of PRPF8 to the speckles of RP13 and equivalent Cas9 controls. The inset shows the magnification of the selected region; **B)** Confocal microscopy analyses of RNA-FISH labelling for U5 snRNA (red) in Cajal bodies (anti-coilin, green) in control and RP13-RPE cells. Arrows indicate CBs. Scale bar is 10 µm; **C)** Immunostaining of iPSCs, KiOs, RPE and ROs with SC35 (SRSF2), a marker for nuclear speckles, showing dispersion of nuclear speckles (white arrows) only in RP13-photoreceptor and RPE cells. Scale bar is 10 µm. **D)** Docking of the U6ISL and U6/5'SS to PRPF8 and the location of crosslinked U6 nucleotides (U64-G65) in the structure of Bact spliceosome. The PRPF8 domain organisation is shown at the top and similar colour codes have been used in the PRPF8 3D structure. The U6 ACAGA-box is also labelled. NTD, N-terminal domain; NTDL, NTD linker; HB, helical bundle; RT, reverse transcriptase-like; En, endonuclease-like; RH, RNase H-like domain. **E)** Profiles of the U5, U4 and Us snRNAs across the gradients shown in **Figure 2C**.

A

Retinal Organoids

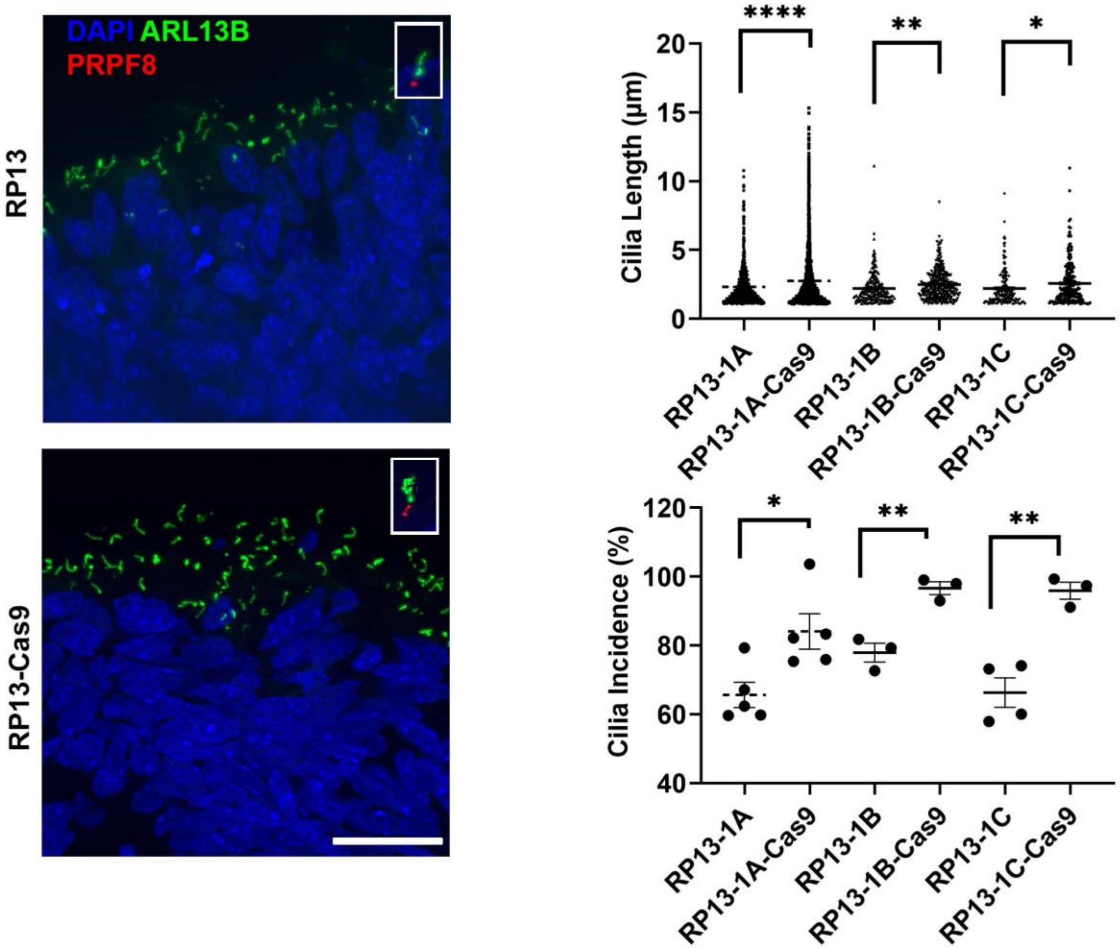

B

Kidney Organoids

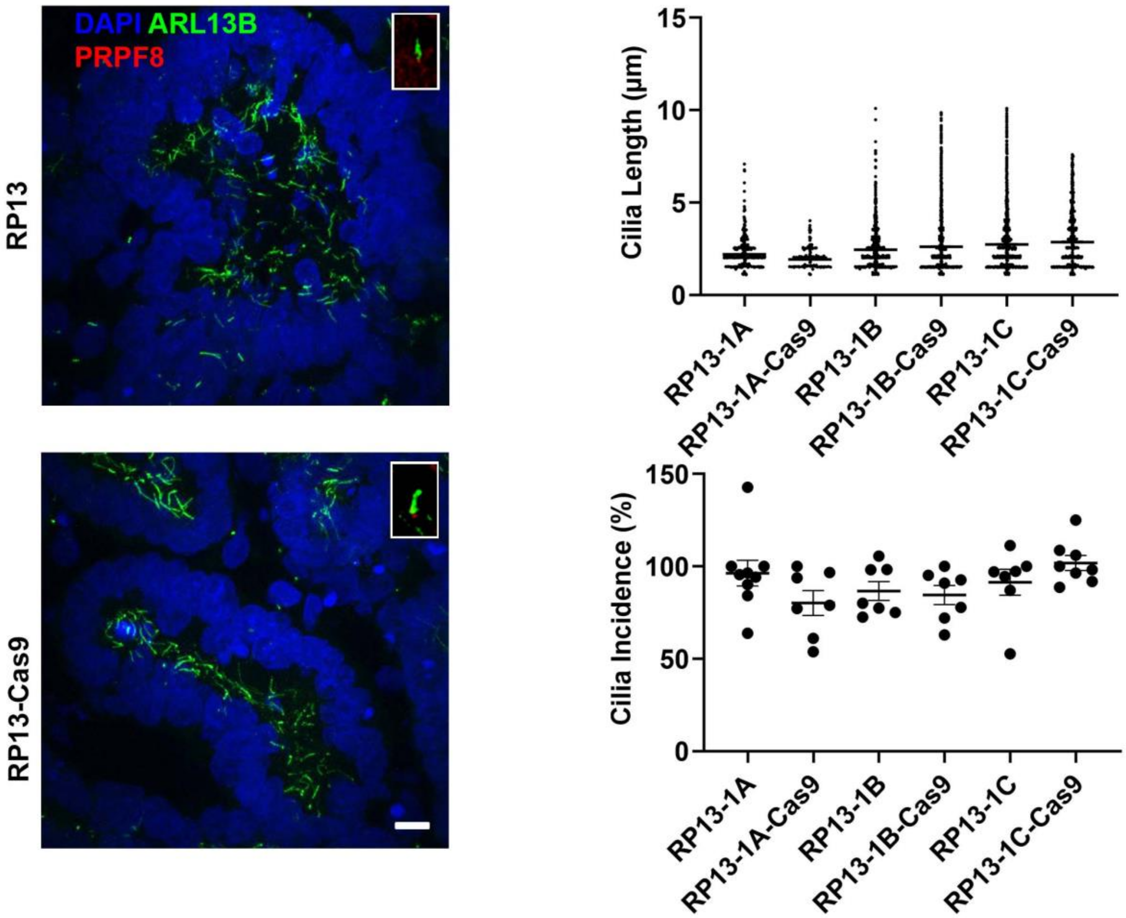

Figure S8. Cilia analysis in ROs and KiOs. A) Immunofluorescent staining of ROs sections with the ciliary membrane marker ARL13B (green) and PRPF8 (red; scale bar is 20 µm). Insets show localisation of PRPF8 to the base of cilia. Graphs show decreased cilia length in RP13 RO in comparison to isogenic controls, as well as decreased cilia incidence. Data shown as mean ± SEM. n = 4993. Statistical significance was assessed using two-tailed paired Student's t-tests (results are indicated using * = <0.05, ** = <0.01, *** = <0.001, **** = <0.0001); **B)** Immunofluorescent staining of KiOs sections stained with ARL13B (green) and PRPF8 (red; scale bar is 10 µm). Graphs show no differences in cilia length and incidence in RP13 KiO in comparison to isogenic controls. Data shown as mean ± SEM. n = 7845. Statistical significance was assessed using two-tailed paired Student's t-test.

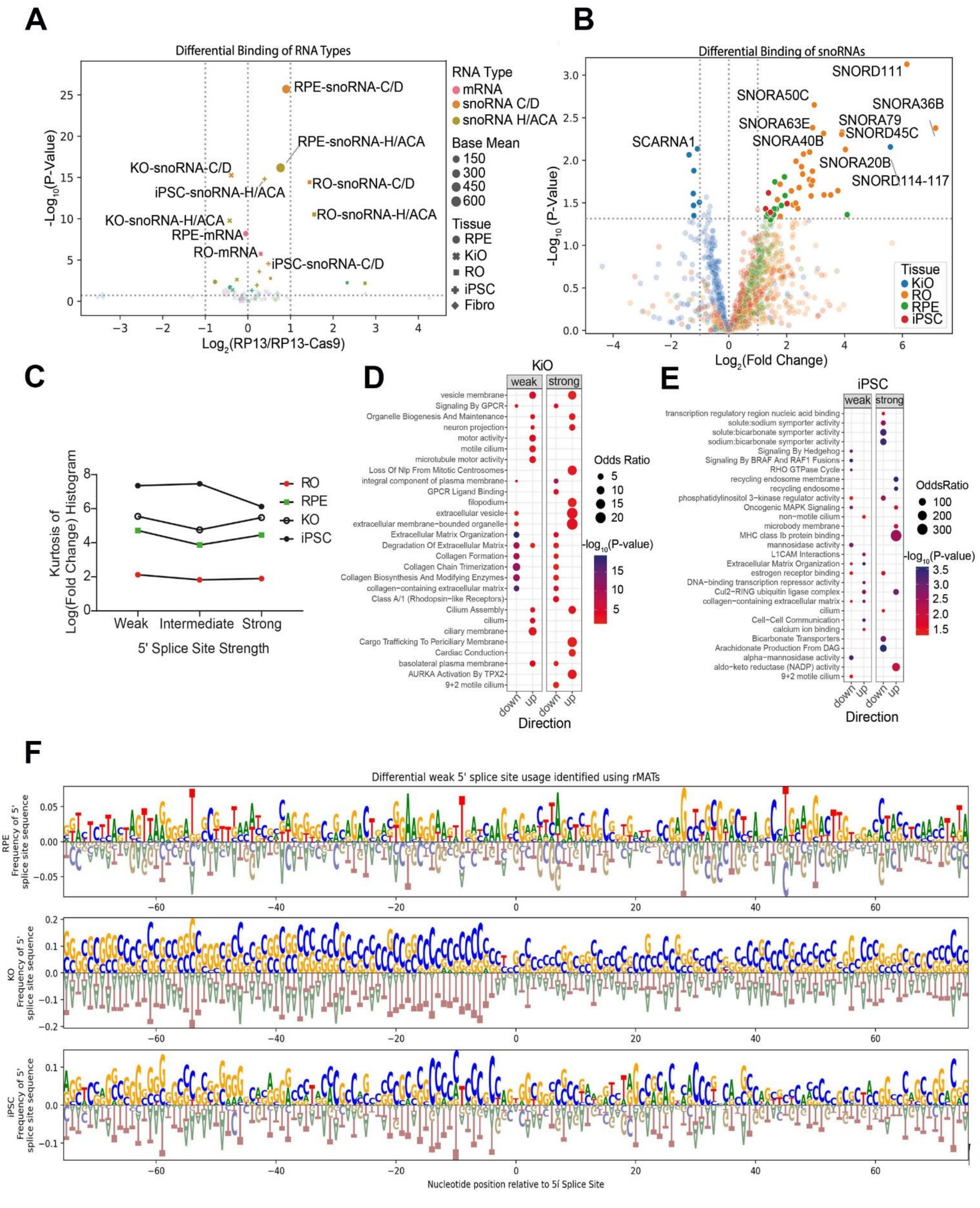
A
Differential Binding of RNA Types
RPE-snoRNA-C/D
RPE-snoRNA-H/ACA
KO-snoRNA-C/D
iPSC-snoRNA-H/ACA
RO-snoRNA-C/D
RO-snoRNA-H/ACA
KO-snoRNA-H/ACA
RPE-mRNA
RO-mRNA
iPSC-snoRNA-C/D
-Log10(P-Value)
Log2(RP13/RP13-Cas9)
RNA Type
mRNA
snoRNA C/D
snoRNA H/ACA
Base Mean
150
300
450
600
Tissue
RPE
KiO
RO
iPSC
Fibro
B
Differential Binding of snoRNAs
SNORD111
SNORA50C
SNORA36B
SNORA63E
SNORA79
SNORD45C
SCARNA1
SNORA40B
SNORA20B
SNORD114-117
-Log10(P-Value)
Log2(Fold Change)
Tissue
KiO
RO
RPE
iPSC
C
Kurtosis of Log(Fold Change) Histogram
RO
RPE
KO
iPSC
Weak
Intermediate
Strong
5' Splice Site Strength
D
KiO
weak
strong
vesicle membrane
Signaling By GPCR
Organelle Biogenesis And Maintenance
neuron projection
motor activity
motile cilium
microtubule motor activity
Loss Of Nlp From Mitotic Centrosomes
integral component of plasma membrane
GPCR Ligand Binding
filopodium
extracellular vesicle
extracellular membrane-bounded organelle
Extracellular Matrix Organization
Degradation Of Extracellular Matrix
Collagen Formation
Collagen Chain Trimerization
Collagen Biosynthesis And Modifying Enzymes
collagen-containing extracellular matrix
Class A/1 (Rhodopsin-like Receptors)
Cilium Assembly
cilium
ciliary membrane
Cargo Trafficking To Periciliary Membrane
Cardiac Conduction
basolateral plasma membrane
AURKA Activation By TPX2
9+2 motile cilium
down
up
Direction
Odds Ratio
-log10(P-value)
E
iPSC
weak
strong
transcription regulatory region nucleic acid binding
solute:sodium symporter activity
solute:bicarbonate symporter activity
sodium:bicarbonate symporter activity
Signaling By Hedgehog
Signaling By BRAF And RAF1 Fusions
RHO GTPase Cycle
recycling endosome membrane
recycling endosome
phosphatidylinositol 3-kinase regulator activity
Oncogenic MAPK Signaling
non-motile cilium
microbody membrane
MHC class Ib protein binding
mannosidase activity
L1CAM Interactions
Extracellular Matrix Organization
estrogen receptor binding
DNA-binding transcription repressor activity
Cul2-RING ubiquitin ligase complex
collagen-containing extracellular matrix
cilium
Cell-Cell Communication
calcium ion binding
Bicarbonate Transporters
Arachidonate Production From DAG
alpha-mannosidase activity
aldo-keto reductase (NADP) activity
9+2 motile cilium
down
up
Direction
OddsRatio
-log10(P-value)
F
Differential weak 5' splice site usage identified using rMATs
RPE Frequency of 5' splice site sequence
KO Frequency of 5' splice site sequence
iPSC Frequency of 5' splice site sequence
Nucleotide position relative to 5í Splice Site

Figure S9

Figure S9: *PRPF8* p.H2309P mutations alters interaction of PRPF8 with snoRNAs and affects 5'SS recognition. A) RP13 mutations affect interaction of PRPF8 with small nucleolar RNAs (snoRNAs). snoRNAs are noncoding RNAs processed from introns in higher mammals that occur in two classes, box C/D and H/ACA snoRNAs. Both classes were significantly affected by *PRPF8* mutation as shown in the volcano plot; **B**) Volcano plot showing how binding of individual snoRNA transcripts are affected by RP13 mutation across all tissues (KiOs = blue, ROs = orange, RPE = green, and iPSCs = red). Statistical significance was assessed using paired t-tests; **C**) Kurtosis of the histograms presented in figure 7B; **D, E**) Gene set enrichment analysis of differentially bound weak and strong 5'SSs in KiOs (D) and iPSCs (E). Direction down and up refers to positive and negative fold changes in (RP13/RP13-Cas9), respectively; **F)** Gene sequence plot showing differential inclusion of nucleotides at weak 5'SSs with upregulated or downregulated binding to PRPF8 in RPE, KiO and iPSCs.

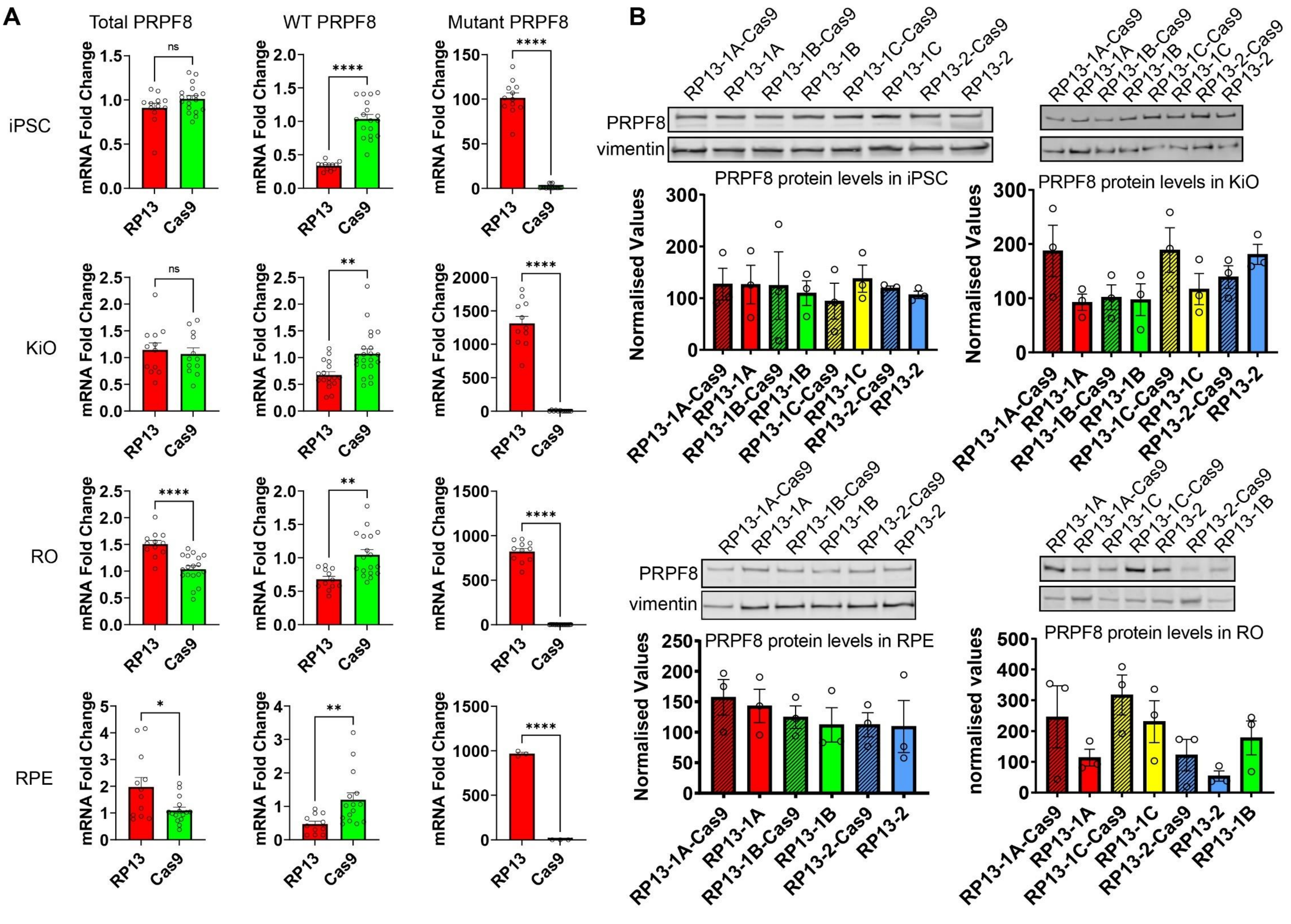
A
Total PRPF8
WT PRPF8
Mutant PRPF8
iPSC
KiO
RO
RPE
mRNA Fold Change
RP13
Cas9
ns
**
****
*
B
PRPF8
vimentin
PRPF8 protein levels in iPSC
PRPF8 protein levels in KiO
PRPF8 protein levels in RPE
PRPF8 protein levels in RO
Normalised Values
normalised values
RP13-1A-Cas9
RP13-1A
RP13-1B-Cas9
RP13-1B
RP13-1C-Cas9
RP13-1C
RP13-2-Cas9
RP13-2

Figure S10: Transcript and protein expression of PRPF8. A) Quantitative PCR (qPCR) data showing effect of RP13 mutation on *PRPF8* mRNA expression across fibroblasts, iPSCs, KiOs, ROs, and RPE cells. Bar charts show the mean mRNA fold change of *PRPF8* in RP13 derived cells relative to the paired RP13-Cas9 controls. n =11 for RP13 and n=16 for RP13-Cas9 samples used for wild type, total and mutant *PRPF8* quantification in iPSCs; n=11 for RP13 and n=11 for RP13-Cas9 samples used for quantification of the mutant and total *PRPF8* transcript in KiOs, n=21 in RP13 and n=16 for RP13-Cas9 samples used for the quantification of wild type *PRPF8* transcript in KiOs; n=11 for RP13 and n=16 for RP13-Cas9 samples used for wild type, total and mutant *PRPF8* quantification in ROs; n=3 for RP13 and n=3 for RP13-Cas9 samples used for mutant *PRPF8* quantification in RPE cells, n=11 for RP13 and n=14 for RP13-Cas9 samples used for total *PRPF8* quantification in RPE cells, n=10 for RP13 and n=14 for RP13-Cas9 samples used for wild type *PRPF8* quantification in RPE cells. Statistical significance was assessed using a paired t-test (* = <0.05, ** = <0.01, *** = <0.001, **** = <0.0001). **B)** Western blot results showing effect of RP13 mutation on PRPF8 protein expression across fibroblasts, iPSCs, RPE, ROs, and KiOs. Bar charts show the mean (n = 3) expression of PRPF8 after normalisation to vimentin (VIM) expression. Statistical significance was assessed using 1-way ANOVA, no significant differences were observed.

Supplementary data 1: **Summary of clinical data of RP13 patients involved in this study.** The sequences of gRNA, ssODN CRISPR/Cas9 used for the *in-situ* gene correction and off-target sequences, and the primers used for amplifying wild type and mutant PRPF8 sequences are also included.

Supplementary data 2: Single-cell RNA-Seq data delineating the expression of highly and differentially expressed genes for each cell cluster in RP13 and RP13-Cas9 ROs. Genes that are differentially expressed between RP13 and RP13-Cas9 rod and cone photoreceptors are listed on separate sheets. The Wilcoxon Rank Sum, two-sided test test was used, the adjusted p values (corrected for multiple comparisons) are shown in the p_val_adj column).

Supplementary data 3: Differential gene expression between RP13 and RP13-Cas9 isogenic controls as determined using DESeq2. The summary sheet shows the number of aligned reads and the percentage of each RNA biotype within the dataset. Four tissue-specific sheets show differential gene expression in iPSCs, KiOs, RPE cells, and ROs. The Wilcoxon Rank Sum, two-sided test test was used, the adjusted p values (corrected for multiple comparisons) are shown in the p_val_adj column).

Supplementary data 4: Summary of GO terms for biological processes, cellular components, and molecular functions in differentially expressed genes from iPSCs, KiOs, RPE cells, and ROs from all RP13 and RP13-Cas9 control tissues. One-sided Fisher's Exact Test with p-value adjustment for multiple comparisons (Benjamini & Hochberg) was carried out.

Supplementary data 5: Differential exon usage between RP13 and RP13-Cas9 isogenic controls. The summary sheets show the number of significantly different splicing events as determined by rMATs (p_{adj}<0.05 and inclusion difference >5%). Alternative splice events (ASEs) are either skipped exon (SE), mutually exclusive exon (MXE), alternative 5'SS (A5SS), alternative 3'SS (A3SS), or retained intron (RI). The number of significant ASEs in RPE (497 genes) and ROs (1500

genes), were greater than those in KiOs (372 genes) and iPSCs (386 genes). Four tissue-specific sheets show differential exon usage (p<0.05 and inclusion level difference > 0.05) in iPSCs, KiOs, RPE cells, and ROs. One-sided likelihood-ratio test with p-value adjustment for multiple comparisons was carried out.

Supplementary data 6: Summary of GO terms for biological processes, cellular components, and molecular functions in genes with differential exon usage from iPSCs, KiOs, RPE cells, and ROs from all RP13 and RP13-Cas9 control tissues. One-sided Fisher's Exact Test with p-value adjustment for multiple comparisons (Benjamini & Hochberg) was carried out.

Supplementary data 7: Cryptic splice event (CSE) site usage between RP13 and RP13-Cas9 isogenic controls. A greater number of significant CSEs were determined by rMATs (p_{adj}<0.05 and inclusion difference >5%) in RPE (205) and RO (106), compared to iPSCs (37) and KiO (57). Four tissue-specific sheets show differential exon usage (p<0.05 and absolute $\log_2$ fold change > 1; modified Wald test and Benjamini & Hochberg for multiple testing adjustment) in iPSCs, KiOs, RPE cells, and ROs. Enrichment analyses of these gene lists are on separate sheets and include GO terms for biological processes, cellular components, and molecular functions as well as reactome terms using Fishers exact test for null hypothesis testing and Benjamini & Hochberg for adjusting for multiple testing.

Supplementary data 8: Transcripts that exhibit differential binding in PRPF8 iCLIP results between RP13 and RP13-Cas9, as determined using DESeq2. Four tissue-specific sheets show differential gene expression in iPSCs, RPE cells, KiOs and ROs with DEseq using a modified Wald test and Benjamini & Hochberg for multiple testing adjustment.

Supplementary data 9: Enrichment analysis of differentially bound transcripts identified using PRPF8 iCLIP. Transcripts were categorised according to maxent5 score into weak, intermediate, and strong splice sites prior to enrichment analysis. Enrichment analyses of these gene lists are on separate sheets for iPSCs, RPE cells, KiOs and ROs and include GO terms for biological processes, cellular components, and molecular functions as well as reactome terms using Fishers exact test for null hypothesis testing and Benjamini & Hochberg for adjusting for multiple testing. Individual differentially bound transcripts in ROs were clustered in specific multiprotein complexes such as RNA polymerase II (POLR1B, POLR1E, POLR2A, POLR2B, and POLR2K), IFT-B (IFT27, IFT88, DYNC1H1, and DYNC2H1) and the BBSome (BBS1, BBS4, and ARL6). In RPE, individual transcripts were for rRNA processing (WDR3, WDR36, WDR43, NOP14), IFT-B (DYNC2H1, TTC26, and KIF3B) and the BBSome (IQCB1, BBS5, CCT2, CCT4). individual differentially bound transcripts encoded ciliary proteins for both iPSCs (IFT57, IFT88, CEP89) and KiOs (WDR35, CLUAP1, DYNC2H1).

Supplementary data 10: Differences in protein expression between RP13 and RP13-Cas9 derived ROs, RPE, and KiOs, as determined using TMT-labelling and mass spectrometry. Proteins (represented by 2 or more unique peptides) are listed with differential expression (DE) between RP13 and RP13-Cas9 control proteomes of ROs (4535 unique proteins, DE=203), RPE (4574 unique proteins, DE=56) and KiOs (4294 unique proteins, DE=4). GO and KEGG enrichment analyses of these protein lists are presented in separate sheets. Two additional sheets compare RO

and RPE protein expression changes with changes in transcript expression. Two-sided t-test was used for statistical analysis.

Supplementary Text: Methods

Derivation and characterisation of RP13-iPSCs

We ascertained three related RP type 13 patients with a *PRPF8* c.6926A>C (p.H2309P) heterozygous missense mutation and one patient with a *de novo PRPF8* c.6926A>C heterozygous mutation[1] (**Supplementary data 1**). All patients were characterised by night blindness in the first decade of life. Dermal skin fibroblasts were reprogrammed to iPSCs using a non-integrative RNA-based Sendai virus system (**Figure S1A**), as previously described[2]. Several clones from each RP13 participant were tested for expression of pluripotent markers (**Figure S1B**), clearance of transgenes (**Figure S1C**) and genome stability (**Figure S1D**). Apart from clone RP13-1A1, which displayed a decreased copy number in the Chr 12p region, all the other clones displayed the expected copy number in the loci that are most susceptible to changes in pluripotent stem cells. Based on these data, one iPSC clone from each participant was selected for further studies and referred to as RP13-1A, 1B, 1C, and 2 as shown in **Supplementary data 1**. All iPSCs were maintained in Matrigel-coated plates with mTeSR™ Plus media and passaged using Versene (EDTA 0.02%) in 1:3 -1: 6 ratio. All cultures were maintained at 37°C, in a humidified environment, with 5% CO_2. Cells were cryopreserved with freezing media containing 90% foetal bovine serum (FBS), 10% dimethyl sulfoxide and 10 µM Y26732.

Sendai Virus Clearance assay

Total RNA was extracted from 5×10^6 iPSCs using TRIzol® reagent following the manufacturer's instructions. Reverse transcription was carried out using 1 µg of total RNA and the SuperScript cDNA Synthesis Kit following the manufacturer's instructions. Polymerase chain reaction (PCR) was carried out using the cDNA from the reverse transcription reaction with GoTaq DNA polymerase. The PCR products were analysed using 2% agarose gel electrophoresis. All primer details are shown in **Table S1**.

Detection of pluripotency markers by flow cytometry

iPSCs were dissociated by incubation with Accutase for 5 minutes at 37℃. A total of $1x10^6$ single cells were resuspended in PBS supplemented with 5% fetal calf serum (FCS) and stained with anti-TRA-1-60 antibody (directly conjugated to FITC, 1:50) and anti-NANOG antibody (directly conjugated to AlexFluor647 conjugate, 1:50) for two hours at 2-8°C followed by PBS washing and subjected to flow cytometry analysis using FACSCanto flow cytometer (BD Biosciences). At least 10,000 cells were acquired, and the data was analysed using the FACSDiva software.

PluriTest analysis

100 ng total RNA extracted from each RP13 and isogenic control iPSCs was used to prepare the GeneChip® for the PluriTest™ analysis to generate a Pluripotency and Novelty Score. The Pluripotency Score is based on many samples (pluripotent, somatic, and tissues) in the stem cell model matrix which consists of an extensive reference set of >450 cell/tissue types, including 223 hESC lines, 41 iPSCs, somatic cells, and tissues. Samples with positive Pluripotency Scores are more similar to pluripotent samples in the model matrix than to all other classes of samples. The Novelty Score is based on well-characterised iPSCs in the stem cell model matrix.

Genome stability assays

Genome stability was assessed using the hPSC Genetic Analysis kit or the Karyostat assay (Thermofisher).

hPSC Genetic Analysis Kit:

Genomic DNA from the patient iPSCs was harvested using genomic DNA extraction kit. Genomic DNA was mixed with 2x qPCR master mix and ROX reference dye for qPCR analysis. Nine pairs of primers were applied for the examination of Chromosome regions 1q, 4p, 8q, 10p, 12p, 12q, 18q,

20q and Xp. qPCR $\Delta\Delta C_t$ values were normalised to normal control iPSCs. A copy number < 1.8 or > 2.2 with a *p*-value <0.05 may indicate the presence of a cytogenetic abnormality.
Karyostat Assay:
To verify the genome stability of CRISPR/Cas9 isogenic controls, 250ng total genomic DNA extracted from each cell line was used in GeneChip® for KaryoStat™ assays for assessment of potential copy number variants or single nucleotide polymorphisms across the genome. The KaryoStat™ assay allows digital visualisation of chromosome aberrations with a resolution similar to G-band karyotyping. The size of structural aberration that can be detected is >2Mb for chromosomal gains and >1Mb for chromosomal losses. The KaryoStat™ array is optimised for balanced whole-genome coverage with a low-resolution DNA copy number analysis. The assay covers all 36,000 RefSeq genes, including 14,000 OMIM® targets, allowing the detection of aneuploidies, submicroscopic aberrations, and mosaic events.

Correction of *PRPF8* p.H2309P mutation in RP13-iPSCs using CRISPR/Cas9
To fully understand the impact of PRPF8 mutation in RPE and retinal cells, CRISPR/Cas9 genome editing was used to correct the PRPF8 c.6926A>C genetic mutation in all patient iPSCs as previously described[2]. For *in situ* gene correction, a guide RNA was designed to include the PRPF8 c. 6926A>C mutation in the 3' region as well as an asymmetric ssODN template with wild-type PRPF8 sequence with the PAM sequence silenced to prevent repeated cleavage by Cas9 (**Figure S2A**). 100 iPSC clones from each patient were selected and tested by restriction site analysis using ApaL1, which cleaves the wild-type sequence "GTGCAC". Approximately 20% of the clones showed complete digestion by ApaL1 (**Figure S2B**): these were further analysed by Sanger sequencing, which demonstrated the correction of the heterozygous mutation "A/C" to homozygous wild type "A" (**Figure S2C**). To exclude possible off-targeting effects, we tested five candidate genomic regions containing sequences partially matched to the gRNA (**Table S1**). All the candidate regions showed DNA sequences that match the wild type without insertion or deletion, indicating that no off-targeting occurred during the CRISPR/Cas9 genome editing procedure (**Figure S2D**). KaryoStat™ analysis, which assesses copy number variants and single nucleotide polymorphisms across the genome (**Figure S2E**) revealed the lack of genomic instabilities in the corrected clones. The CRISPR/Cas9 RP13-iPSC clones displayed high pluripotency scores in the PluriTest™ analysis (**Figure S2F**), confirming their pluripotent phenotype. From herein, unedited iPSCs that harbour the pathogenic c.6926A>C mutation in PRPF8 are collectively referred to as "RP13". Similarly, the edited isogenic controls (c.6926C>A) are referred to as "RP13-Cas9".

Differentiation of iPSCs to RPE Cells
Differentiation was performed as previously described[3]. In brief, RP13 and RP13-Cas9 iPSCs were grown on 6 well plates coated with growth factor-reduced Matrigel™, as recommended by the manufacturer's instructions. Cells were incubated at 37ºC, 5% CO_2 and fed every second day with 2 ml mTeSR™ Plus per well. Once iPSCs achieved 80-95% confluency, mTeSR™ Plus media was replaced with 3 ml of differentiation medium (DMEM/F-12, 50 µM β-mercaptoethanol, 1X MEM NEAA, 1X penicillin streptomycin, 20% knockout serum replacement and 10 mM nicotinamide). Differentiation media was partially changed every second day. On days 8 and day 14 media composition was adjusted, by omitting nicotinamide and adding 100 ng/ml Activin A or 3 µM CHIR99021, respectively (**Figure 1A**). RPE patches formed during this time and were manually excised using a scalpel and transferred to a Falcon tube containing differentiation media. Next, media was aspirated, and patches were washed quickly with PBS.

Then patches were dissociated by incubation at 37°C with TryplE Select (10X) for approximately 20 minutes followed by passage through a 40 µm cell strainer. TryplE Select was neutralised with RPE media and cells were centrifuged for 5 minutes at 300 x *g*. Media was aspirated and replaced with fresh RPE maintenance media (DMEM/F-12, 50 µM β-mercaptoethanol, 1X MEM NEAA, 1X

penicillin streptomycin, and 4% knockout serum replacement). iPSC-RPE were counted using a haemocytometer and plated at density of $1.5x10^5/cm^2$ on 6 well plates coated with growth factor-reduced Matrigel™ in 3 ml RPE maintenance media. Media was partially replaced every 2-3 days until cells were pigmented and had a typical "cobblestone" epithelial cell morphology. Cells were then dissociated using TryplE Select (10X) and plated onto 24-well and 12-well PET 0.4 µm pore Transwell ThinCerts™.

Immunofluorescent Staining of iPSC-RPE cells

iPSC-RPE cells grown on 24-well ThinCerts™ were washed with PBS and incubated with 4% paraformaldehyde (PFA) for 20 minutes at room temperature. PFA was then removed, cells were rinsed twice with PBS and stored in PBS at 4°C until required. The transwell culture surface was isolated and placed on Superfrost+ microscope slide. Pigmentation of RPE cells was removed using Melanin Bleach Kit, as recommended by the manufacturer. RPE cells were blocked in PBS supplemented with 10% normal donkey serum and 0.3% Triton-X100, for 1 hour at room temperature. For ezrin immunostaining, RPE cells were not bleached, and Triton X-100 was substituted for a 5-minute incubation at -20°C with methanol, prior to blocking. The RPE cells were incubated with primary antibodies overnight at 4°C. Following three washing with PBS, RPE cells were incubated with secondary antibodies diluted in antibody dilution (PBS and 1% normal donkey serum) with Hoechst for 1 hour at room temperature. Then, iPSC-RPE cells were washed 3X for 5 minutes with PBS before mounting with Vectashield and sealed with a #1 coverslip. RPE cells were imaged using an AxioImager upright microscope with Apotome structured illumination fluorescence using 20x, 40x and 63x oil objectives (Zeiss, Germany). Three to five images per patient were processed using ZEN 2.5 Lite software (Zeiss). For cilia studies, RPE cells were imaged using an 100x objective on an A1R confocal microscope (Nikon). Images were analysed using Fiji (ImageLab) to assess co-localisation of ciliary proteins, and Huygens software (Scientific Volume Imaging) to determine cilia length. Data were analyzed and processed in Prism 7 (GraphPad).

Measurement of trans-epithelial resistance (TEER)

Trans-epithelial resistance (TEER) was measured in Transwells containing RPE cells by inserting the tip of the short electrode in the inner side of the Transwell insert, and the tip of the longer electrode in the outer side of the Transwell insert. Also, the resistance of a blank TW containing PBS was measured. Three measurements were obtained from each Transwell insert. After that, the readings from the blank Transwell were subtracted from the readings obtained from Transwells containing RPE cells. Based on the formula Unit area resistance = Resistance (Ω) x Effective Membrane Area (cm2), the results were multiplied by 0.3, which is the membrane area of a Transwell, and final values were given in ohms (Ω). TEER measurements were performed one day after feeding the RPE cells to avoid variability in the TEER readings.

Phagocytosis assay

Bovine rod photoreceptor outer segments (POSs) were centrifuged at 2600 × *g* for 4 minutes, and the pellet was resuspended with 0.4 mg/ml FITC for 1 hour at room temperature followed by agitation in the dark. Following centrifugation at 2600 × *g* for 4 minutes and washing of the POSs three times with PBS, POSs were resuspended in AdRPMI 1640 media supplemented with B-27 Supplement and FBS. The staining of POSs was confirmed under a Bioscience Axiovert microscope. RPE cells were treated with unlabelled and FITC-labelled POSs (20 POSs per cell) and incubated for 4 hours at 37°C. For control, RPE cells were treated with FITC-labelled POSs and were incubated at 4°C for 4 hours. Both control and RP13-RPE cells were washed with PBS and were detached from the wells using 200µl of TryplE Select (10X) for 15 minutes. Then, control and RP13-RPE cells were resuspended in flow buffer (PBS with 2% FBS) and were transferred into Eppendorf tubes followed by centrifugation at 300 × g. To distinguish cells from debris and unbound POSs, RPE cells were incubated with 5 mM DRAQ5 for 5 minutes. To quench fluorescence from unbound POSs, cells

were incubated with 0.2% Trypan Blue solution for 10 minutes. RPE cells were washed 3 times with PBS, and cell pellets were resuspended in flow buffer. Samples were run on a LSRII flow cytometer, and 10,000 events were collected per sample. Results were analysed using FacsDiva software.

RPE cytokine secretion studies

Medium from basal and apical chambers of transwell inserts were collected from RPE cells of healthy controls and patients. The levels of PEDF and VEGF secretion were measured by using human PEDF-ELISA Kit (Cusabio, CSB-E08818h) and human VEGF-ELISA Kit (Life Technologies) according to the manufacturer's instructions.

ROs differentiation from iPSCs

7000 iPSCs were seeded into each well of Lipidure-coated 96-well U-bottomed plate and cultured for 48 hours to form Embryonic Bodies before start of differentiation. From day 0 to day 18, the differentiation medium was added into the culture containing 41% IMDM, 41% HAM's F12, 15% KOSR, 1% GlutaMAX, 1% Chemically defined, lipid concentrate, 1% Pen/Strep, 450 μM (20 μl of stock) 1-Thioglycerol. 1.5nM BMP4 was added from day 6 to day 9 (**Figure 1D**). From day 18, the culture medium was changed to 10% FBS in DMEM/F12 supplemented with 1x N2, 0.1mM taurine and 0.5 μM retinoic acid until day 150.

Immunofluorescent Staining of iPSCs-ROs

10 μm thickness sections of ROs were collected on glass cover slips and were separated using an ImmEdge pen. Sections were air-dried for 20 minutes at room temperature and washed with PBS to remove OCT 91 from the tissue. ROs sections were incubated with a blocking solution (5% normal goat or donkey serum, 0.3% Triton-X in PBS) for 1 hour at room temperature, followed by overnight incubation with primary polyclonal non-conjugated antibodies shown in the Key Resources Table at 4°C. Primary antibodies were diluted in antibody diluent solution containing PBS, 1% bovine serum albumin (BSA) and 0.3% Triton-X. Following overnight incubation, retinal sections were washed three times with PBS for 15 minutes each and incubated with secondary antibodies conjugated to fluorophores and diluted in antibody diluent for 1 hour at room temperature. RO sections were then washed three times with PBS for 10 minutes each and covered by Hoechst nuclear stain diluted in Vectashield at 1:2000, followed by mounting of the slides by a coverslip.

KiOs differentiation from iPSCs

Kidney cell and organoid culture was based on established protocols[4]. RP13- and RP13-Cas9-iPSCs were cultured in mTeSR™ Plus. To start differentiation into kidney cells, iPSCs were dissociated into single cells with TrypIE. 400,000 cells were plated in Matrigel-coated 6-well plate with 2 ml mTeSR™ Plus containing 10 μM ROCK inhibitor (Y-27632) and incubated overnight (day -1). The next day, cells were at 50% confluency and media was changed to APEL2 containing 5% protein-free hybridoma medium (PFHM-II) (complete medium; henceforth called APEL2+) and 8 μM CHIR99021 (day 0). The same media change was done on day 2. On day 4 of differentiation, media was changed to APEL2+ containing 200 ng/ml FGF9 and 1 μg/ml heparin. Media was changed every other day. On day 7 of differentiation, kidney cells were trypsinised, resuspended into a single cell suspension and counted. 500,000 cells were transferred to an Eppendorf tube and spun down at 800 x *g* for 3 minutes at room temperature three times, with gentle tube agitation after each spin. 3D KiO cultures were grown on an air-liquid interphase, on cell culture inserts called Thincerts (Greiner Bio-One Ltd). Kidney cell pellets were transferred into the inner part of the Thincert, and KiOs were allowed to mature. APEL2+ containing 5 μM CHIR99021 was added to the outer part of the Thincert and incubated for 1 hour at 37°C, following media change to APEL2+ containing 1x antibiotic/antimycotic (complete media; henceforth called APEL2++), 200 ng/ml FGF9 and 1 μg/ml heparin. Media containing growth factors was changed every other day throughout the

differentiation. Growth factors were withdrawn on day 12 and culture with APEL2++ media was carried on until day 25.

Cyst induction

A 24-well plate was coated with Lipidure by placing 500 µl of 0.5% w/v Lipidure solution in each well and allowing complete evaporation at room temperature in sterile conditions. On day 22, KiOs were moved from Thincerts into a non-adherent plate with APEL2++ media containing 30 µM forskolin, 12.5 µM blebbistatin or DMSO. Organoids were grown in suspension with drugs for 72 hours.

KiOs processing

On day 25 organoids were washed with PBS and snap-frozen in liquid nitrogen for biochemical and proteomic assays. Organoids for RNA sequencing were suspended in 1 ml Trizol and snap-frozen. Organoids for immunofluorescent staining were fixed with 4% PFA for a minimum of 4 hours, followed by three PBS washes. They were permeabilized and blocked using 2% BSA in PBS containing 0.3% Triton X-100 overnight at 4°C. After three PBS washes, organoids were incubated in primary antibodies diluted in 1% BSA overnight at 4°C. Following three PBS washes, organoids were incubated in secondary antibodies diluted in 1% BSA overnight at 4°C. On the final day, organoids were washed three times with PBS and imaged with a confocal A1R microscope (Nikon) at x4 magnification. KiOs for OCT embedding were fixed with 4% PFA, washed with PBS and soaked in 30% (w/v) sterile sucrose overnight at 4°C on a roller. Following a PBS wash, organoids were placed in moulds, submerged in OCT and frozen. Frozen OCT blocks were cryosectioned at 10 µm thickness on a Leica CM1950 cryostat. Sectioned organoids were stored at -80°C until use. Further processing is described below. Images were processed in Fiji (ImageJ). Image processing included the use of the following plugins: “Colocalisation Threshold,” “CiliaQ” and “Mitochondria Analyser.”

Transmission Electron Microscopy (TEM)

Trans-wells of RPE cells, ROs and KiOs were washed with PBS and fixed overnight with 2% glutaraldehyde in 0.1 M sodium cacodylate buffer at 4°C. The samples were post-fixed in 1% osmium tetroxide, dehydrated in an acetone gradient, and embedded in epoxy resin. Ultrathin sections of 70 nm thickness were picked up on copper grids, stained with uranyl acetate and lead citrate and imaged using a Philips CM100 Hitachi HT7800 transmission electron microscope with high-resolution digital image capture. For iPSC-RPE, ten cuboidal cells were images per sample at a magnification of 5000x. Sample identifications were created using a random number generator, images were then sent to researchers who were blinded to *PRPF8* mutation status. Melanosomes and vacuoles were counted manually by first importing the images into Microsoft PowerPoint. All other measurements were taken using Microscopy Image Browser (MIB)[5].

Cilia Length and Frequency Measurements in RPE Cells, ROs, and KiOs

Samples were blocked in 1% non-fat milk in PBS for 30 minutes then stained with ARL13B primary antibody. Images were obtained using a Nikon A1R confocal microscope with ×100 oil objective lens controlled by NIS Elements AR 4.20.01 (Nikon) software. Optical sections were generated through structured processing using NIS Elements AR 4.20.01 (Nikon) software. Images were deconvoluted using the deconvolution wizard and batch processor in Huygens software (Scientific Volume Imaging). ARL13B objects were segmented automatically in Huygens and the object analyser was used to calculate ARL13B object length. A minimum of 500 cilia from at least three non-overlapping fields of view were analysed per sample. Nuclei were counted manually, and data was then used to calculate ciliary incidence.

Serial block face scanning electron microscopy (SBF-SEM)

RPE cells were fixed overnight in 2% glutaraldehyde in 0.1M sodium cacodylate buffer. Once fixed, the samples were processed using an adapted heavy metal staining protocol[6]. Briefly, samples were incubated in a series of heavy metal solutions -3% potassium ferrocyanide in 2% osmium tetroxide, 10% thiocarbohydrazide, 2% osmium tetroxide again, 1% uranyl acetate overnight, and finally lead aspartate solution. Between each step the samples were rinsed thoroughly in several changes of deionised water. Samples were dehydrated through an acetone gradient and then impregnated with increasing concentrations of Taab 812 hard resin, with several changes of 100% resin. The samples were flat embedded in 100% resin and left to polymerise at 60°C for a minimum of 36 hours. The resin blocks were trimmed to approximately 0.75 mm by 0.5 mm and glued onto an aluminium pin. To reduce sample charging within the SEM, the block was painted with silver glue and sputter-coated with a 5 nm layer of gold. The pin was placed into a Zeiss Sigma SEM incorporating the Gatan 3view system, which allows sectioning of the block *in situ* and the collection of a series of images in the z-direction. Multiple regions of interest were imaged at x2000 magnification, 3000 x 1500 pixels scan, which gave a pixel resolution of approximately 15 nm. Section thickness was 100 nm in the z-direction. The resulting z-stacks were post processed in MIB using batch processing and cilia were identified and segmented manually using MIB. The segmentations were imported into Amira (FEI) for construction of the 3D models.

Quantitative real-time polymerase chain reaction (RT-PCR)

Total RNA was extracted using the ReliaPrep RNA Cell Miniprep System according to the manufacturer's protocol. One µg of RNA was reverse transcribed into cDNA with random primer by reverse transcriptase (RT). Relative gene expression was determined by quantitative real-time RT-PCR, using 20 ng cDNA (relative to RNA amount) for each sample with the SYBR Green Master Mix in a 10µl reaction. C_t values were measured using QuantStudio™ 7 Flex Real-Time PCR System (ThermoFisher). Primers against *GAPDH* were used as the endogenous control to normalize the amounts of RNA in each sample. Primer sequences are shown in **Table S1**.

RNA extraction and RNA-Seq

Trizol suspensions of iPSCs, RPEs, KiOs and ROs were homogenized using 26-gauge syringe needles followed by standard Trizol RNA extraction. Extracted RNA was further purified using DNAse TURBO, and RNA purity and concentration was evaluated on TapeStation (Agilent Technologies). RNA libraries were prepared using TruSeq Stranded mRNA libraries and a total of 105 samples was run on four lanes of NextSeq 2000 at 200 cycles 100bp paired end (each tissue type was run per lane).

Single cell RNA-Seq

ROs samples at day 210 of differentiation were dissociated to single cells using a neurosphere dissociation kit (Miltenyi Biotech). 10,000 cells from each sample were captured, and sequencing libraries generated using the Chromium Single Cell 3' Library & Gel Bead Kit (version 3.1. 10x Genomics) for scRNA-Seq. Single cell RNA-Seq libraries were sequenced to 50,000 reads per cell on an Illumina NovaSeq 6000. The 8 samples were aligned to human reference genome (GRCh38) using CellRanger Version 3.0.1. Dead cells and debris were removed from downstream analysis by excluding cells with a minimum of 2000 counts per cell, a minimum of 500 genes per cell and a maximum of 20% mitochondrial reads per cell. Doublets were removed using DoubletFinder. A standard Seurat (version 4.3) clustering workflow was followed to identify clusters in each cell. Default parameters were used for the NormalizeData, FindVariableFeatures, ScaleData, RunPCA, and FindNeighbors. Each sample was clustered at a resolution of 1 with the FindClusters function and a UMAP plot using the first 20 principal components was used to visualise the data. To create a combined dataset each sample was downsampled to 5500 to ensure that clustering was not driven by cell number. There were small technical differences between the number of counts and number

of genes and percentage mitochondria between the three samples. These factors were regressed out using the ScaleData function and Harmony batch correction applied. The data was reclustered using the above method. The first 10 principal components were used for FindNeighbors and RunUMAP functions. We tested a range of clustering resolutions from 0.2 – 2.2. Differentially expressed markers for each cluster were obtained using the FindAllMarkers function for each resolution. Cell types were annotated using known marker genes.

Bulk RNA-Seq Data analyses

Quality control and alignment against the reference genome:

RNA paired-end sequencing quality control was assessed using FastQC[7] and multiQC[8] giving on average 76 million mapped reads (**Table S3)**. Both adapters and low-quality bases (QV < 20) were trimmed from read extremities using Trimmomatic[9]. All libraries were mapped against the Human GRCh38 release-105 reference genome (retrieved from http://ftp.ensembl.org/pub/release-105/fasta/homo_sapiens/dna/Homo_sapiens.GRCh38.dna.primary_assembly.fa.gz) through the STAR aligner[10] with default parameters. STAR-generated sorted BAM output files were used for assigning read counts to gene features with featureCounts[11] with the following parameters: -p -B -C -M -O --fraction -s2 and using Homo_sapiens.GRCh38.105.gtf annotation file downloaded from the same Ensembl ftp address as above.

Differential gene expression:

Read counts tables generated by featureCounts and Libinorm[12] were used as input for differential expression (DE) analyses using DeSeq2[13]. DE pairwise comparisons were performed either for each cell type per participant, or for each cell type per pooled participant samples. In all analyses, the fold-change ratio was expressed as mutant RP13: isogenic control RP13-Cas9. The same read count table analyzed for multi-dimensional scaling (MDS) using the plotMDS function in the EdgeR package[14]. EnhancedVolcano (https://bioconductor.org/packages/release/bioc/html/EnhancedVolcano.html) was employed for an overall DE visualization through volcano plots. Tools were run under the R environment, version 4.1.0.

Evaluation of Differential Splicing:

STAR-generated sorted BAM files were used as input for rMATs turbo v4.1.2[15] for differential splicing (DS) analyses under the following parameters: --libType fr-firststrand --readLength 88 --variable-read-length –novelSS. The pairwise comparisons of sample groups for DS analyses followed the same rationale as for DE described above. We wrote a PERL script (https://github.com/eltonjrv/bioinfo.scripts/blob/master/filter_rMATs.pl) to filter rMATs results according to our set of pre-established thresholds: FDR < 0.05, Inclusion Level Difference (IncLevelDifference) > 0.05, and both inclusion and skipping junction counts average (IJC_SJC_avg) > 5. DEXSeq[16] was also used with default parameters for differential exon usage (DEU) score calculations, following the same pairwise comparisons as for both DE and DS analyses. Uniquely mapped, junction-spanning reads were used by MAJIQ to construct splice graphs for transcripts from a custom Ensembl transcriptome annotation, and to quantify PSI and ΔPSI for all local splicing variations (LSVs). The captured LSVs include classical alternative splicing events (e.g. cassette exons, alternative 5'SSs, etc.) as well as more complex variations[17]. In addition, we employed SUPPA[18] and MAJIQ[19] to determine both PSI values (within conditions) and ΔPSI values (between conditions). Regional Coverage was counted using Sailfish quant[12] followed by the tools described above. The differences in variation of were analysed using an f-test for each event, and a t-test was used to compare the log10 normalized value of the whole dataset per tissue. f-tests and t-tests were conducted using custom code written in SciPy[20].

Gene enrichment functional analysis

Gene lists of differentially expressed genes at DE or DS levels were enriched using GSEApy[21], using the Genetic Ontology (GO)[22] and Reactome[23] libraries. GO-based gene enrichment analyses

were performed by clusterProfiler Bioconductor package[24] under R v4.1.0, set to an adjusted *p*-value < 0.05. We limited the background gene set to only include those genes that are expressed in the relevant RNA-seq dataset for each cell type to improve the accuracy of identifying enriched pathways altered between cell types and conditions.

Alphafold2 analysis of predicted protein structures

PRPF8 protein sequence (XP_024306305.1) and mutant versions were folded using ColabFold[25] using an Alphafold2 backend[26], running in single sequence mode (alphafold2_ptm model) and five recycling steps. We generated 25 replicates with differing seeds to average out small folding variations. All models were rotated and translated to fit an arbitrarily chosen model (wildtype with seed 001) before averaging them. Functional regions were obtained from previous mutational studies[27].

TMT labelling for mass spectrometry

Total cell lysates were prepared from RP13 and RP13-Cas9 ROs, KiOs, IPSCs or RPE cells following the instructions of the TMT10plex™ Isobaric Mass Tagging Kit (Thermo Scientific). After pipetting cells in the lysis buffer and heating at 95°C for 5 minutes, lysates were diluted to 120 µl and sonicated. Protein concentrations were determined using the Pierce BCA protein assay kit (Thermo Scientific). To this end, 100 µg proteins from each sample were reduced by the addition of tris(2-carboxyethyl)phosphine, alkylated with iodoacetamide followed by acetone precipitation. Protein pellets were resuspended in 50mM triethyl ammonium bicarbonate (TEAB) buffer and were digested with trypsin overnight at 37 °C. Peptides from each sample were then labelled with a specific TMT reagent for 1 hour at room temperature. Reactions were quenched by 5% hydroxylamine for 15 minutes. Fifty micrograms of TMT-labelled peptides from RP13 and control samples were combined and cleaned up using C18 spin columns (Harvard Apparatus) and subjected to pre-fractionation using the high-pH reversed-phase peptide fractionation kit following the manufacturer's instruction (Thermo Scientific). Collected fractions were dried and resuspended in 50 µl of 0.1% formic acid for mass spectrometry analysis.

LC-MS/MS analysis

Peptides in each fraction were analysed in two injection replicates using either an Orbitrap Fusion or a Q Exactive HF-X mass spectrometer (Thermo Fisher Scientific), both coupled with an UltiMate 3000 RSLCnano HPLC system (Thermo Fisher Scientific), as previously described[2].

Data processing

MS/MS spectra were searched against a SwissProt human database containing 20,341 reviewed protein entries using the MASCOT algorithm (Matrix Science) in Proteome Discoverer 2.2 (PD, Thermo Fisher Scientific), and were processed as previously described[2]. At least two quantifiable unique peptides in each replicate were required for protein quantification. Protein ratios were log-transformed and then median normalised using the Perseus software version 2.0.6.0[28]. The reported RP13: RP13-Cas9 (mutant : control) ratios are the average of two replicates. To identify the differentially expressed proteins, those proteins with a $\log_2$-fold change (LFC) less than −0.5 or greater than +0.5 and a p-value <0.05 were defined as differentially expressed (DE). Gene Ontology (GO) enrichment analyses were carried out by ShinyGo (FDR cut-off 0.05).

Western blot

We have used two methods of Western blot, for PRPF8 protein levels across different tissues we used LI-COR reagents, for the remaining protein levels analysis we used ECL method. Cells were harvested and washed with cold PBS, re-suspended in lysis buffer (25mM Tris-Cl pH 7.5, 120 mM NaCl, 1 mM EDTA pH 8.0, 0.5% Triton X100) supplemented with protease inhibitors (Roche 11697498001) and lysed by ultra-sonication (twice, for 6 seconds) (Bradson Sonifier150) to obtain

whole cell lysate. The protein concentration was determined using the Bradford Dye Reagent (Bio-Rad 500-0205). Fifty µg of whole lysate was applied to SDS-PAGE and transferred to Hybond PVDF membrane (GE Health 15259894), followed by standard western blot procedure. The bound primary antibodies were detected using horseradish peroxidase (HRP)-conjugated secondary antibody and the ECL detection system (GE Health GERPN2232).

Proteins were extracted from cells and organoids using NP40 lysis buffer containing protease inhibitors. Protein extracts were sonicated five times for 5 seconds at 5 mA, with samples kept on ice at all times. Sonicated protein extracts were incubated on a rotator at 4°C for 30 minutes, followed by centrifugation at 15,000 x *g* for 15 minutes at 4°C. Protein concentration was determined using the DC™ Protein Assay Kit II. Ten µg of protein extract was loaded on 3-8% Tris-Acetate gel and run for 1 hour and 20 minutes at 150V. Proteins were then transferred on PVDF membrane overnight at 25V at 4°C. Membrane was blocked in Intercept Blocking Buffer for 1 hour at room temperature and incubated with primary antibodies overnight on a roller at 4°C. After four 1xPBS washes membrane was incubated with secondary antibodies for 1 hour at room temperature in the dark and again washed four times with 1xPBS. After one dH_2O wash, the membrane was dried by sandwiching between two pieces of blotting paper. Dried membranes were then imaged on LI-COR, followed by band intensity calculations.

Analysis of snRNPs Levels by Glycerol Gradient Fractionation and Northern blotting

Analysis of snRNPs levels were performed according to our previous protocol [29] with minor modifications. Briefly, RPE cells were washed with PBS, harvested by centrifugation and pellets were resuspended in 20 mM HEPES-KOH, pH 7.9, 100 mM KCl, 10% (v/v) glycerol, 0.2 mM EDTA, 1 mM DTT. Whole-cell extracts were prepared using syringes with 18-gauge needles followed by sonication (three times bursts at 30% for 5 seconds on ice, and 30 seconds pauses in between bursts). Sonicated lysates were cleared by centrifugation at 15,000 x *g* for 20 minutes at 4°C, and were aliquoted, flash frozen and stored at -80°C. Whole-cell extracts were sedimented on linear 4 ml 10-30% (v/v) glycerol gradients in the G150 buffer (20 mM HEPES pH 7.9, 150 Mm NaCl, 1.5 mM $MgCl_2$ and 0.5 mM DTT). After ultracentrifugation in a Sorvall TH-660 rotor for 14 hours at 29,000 rpm (114136.7 x g), the gradients were fractionated into 24 fractions. To analyse the relative levels of snRNPs, proteins in the odd-numbered gradient fractions were digested with proteinase K in 20 mM HEPES-KOH pH 7.9, 150 mM NaCl, 10 mM EDTA and 1% (w/v) SDS for 45 minutes at 42°C. The total RNAs were extracted with phenol/chloroform/isoamylalcohol and were precipitated with sodium acetate and ethanol at -20°C for 2 hours. The isolated RNAs were separated by denaturing 8% urea PAGE followed by Northern blotting and hybridisation with 5'-end biotinylated probes against U4, U6 and U5 snRNAs[30]. The biotin-labelled probes were detected using the chemiluminescent nucleic acid detection module kit (Thermo Scientific) according to the manufacturer's instructions. To analyse the distribution of selected splicing proteins across the gradients, proteins were acetone precipitated from even-numbered gradient fractions and separated on NuPAGE 4−12% Bis–Tris gels (Invitrogen) followed by Western blotting on a nitrocellulose membrane (GE Healthcare). Immunostaining for PRPF8, Brr2 (SNRNP200), Snu114 and PRPF31 was performed using specific antibodies and the Amersham ECL detection kit (GE Healthcare). All antibody details are shown in the **Key Resource Table**.

Fluorescence *in situ* hybridisation

Fluorescence in situ hybridisation (FISH) was performed using U2, U4, U5 or U6 snRNA probes labelled with AlexaFluor647 or an oligo dT(45) probe labelled with ATTO488 at the 5'-end. The sequences of snRNA probes and the RNA-FISH method were as previously described[29,30]. After FISH, for snRNA probing, cells were immunostained for coilin. For poly-A^+ RNA FISH, cells were immunostained for SC35 and prepared for confocal microscopy as described in the methods

sections named immunofluorescent staining of RPE cells and ROs. Quantification of mean intensities was performed with the ImageJ/Fiji software from three independent measurements.

Co-localisation of SC35 and PRPF8 – Image analysis

Co-localization analysis of SC35 and PRPF8 immunofluorescence images was performed using ImageJ/Fiji software. Each coloured image was separated into individual channels; red (for PRPF8), green (for SC35), and blue (Hoechst). For each dye, the same sized region of interest (ROI) was selected for further analysis. The degree of co-localization was calculated as mean of the correlated pixel intensities of the red and green channels, using the embedded co-localization analysis plug-in at default settings.

iCLIP-seq library preparation

PRPF8–RNA interactions were stabilized by UV crosslinking (254 nm, 400 mJ/cm^2) of cells, followed by lysis in 1 mL of iCLIP lysis buffer (50 mM Tris-HCl, pH 7.4, 100 mM NaCl, 1% Igepal CA-630 (Sigma I8896), 0.1% SDS , 0.5% sodium deoxycholate), limited digestion with RNase I (0.8 units/ml), immunoprecipitation of PRPF8–RNA complexes with anti-PRPF8 antibody at 10 µg per mL of lysate and 150 µl magnetic protein A Dynabeads, followed by stringent washes. After dephosphorylation with FastAP alkaline phosphatase and T4 Polynucleotide Kinase, a barcoded pre-adenylated adaptor (0.1 µM) was ligated to the 3′ end of precipitated RNA using T4 RNA ligase I (3 units/µl). Ligation was performed on-bead in 20% PEG8000 to improve ligation efficiency. Unincorporated adaptor was removed by treatment with 5’ deadenylase (1.25 U/µl), RecJf endonuclease (0.75 U/µl) and stringent washes. PRPF8-RNA complexes were eluted from Dynabeads using preheated 1x NuPAGE loading buffer at 70℃ and loaded onto a 4-12% NuPAGE Bis-Tris gel running for 65 minutes at 180V. After transfer to a nitrocellulose membrane (30V, 2 hours), a region 75 kDa (~220 nt of RNA) above PRPF8 (274 kDa) was excised from the membrane and treated with proteinase K to isolate RNA. The RNA was reverse transcribed by Superscript IV reverse transcriptase using irCLIP_ddRT primers (1pmol/µl) and circularized using CircLigase II. The cDNA was PCR amplified for 21 cycles with Platinum™ II Hot-Start PCR Master Mix using P5Solexa/P3Solexa primers. The PCR product was size selected using agarose gel electrophoresis, and the 150-400bp region was excised for gel extraction followed by purification with AMpure XP. This was then sequenced by using the Illumina sequencing platform (See **Table S1** for oligonucleotide sequences and **Key Resource Table for** catalogue numbers of all reagents).

iCLIP analysis:

FastQC[7] was used for quality control of iCLIP-seq data similar to the raw data from RNA-seq. Reads were mapped using STAR[10] onto GRCh38 and duplicate reads were filtered by UMItools[31]. Events were quantified by assessing the highest peak within annotated regions (GRCh38), rather than RPKM, to identify peak locations without averaging them out. Significant differential events were normalised and quantified using DESeq2[13]. Significant events were annotated by RNA biotype using the RNAcentral database[32]. We assessed splice sites using maxentpy/MaxEntScan[33] to calculate the 3’ and 5’SS strength of exons corresponding to each event, based on the gene annotation in GRCh38.

Mean and Error bar information for Figure 2F

Tissue	Status	Mean	SEM
iPSCs	Control	0.76569	0.26019
KiO	Control	2.7467	1.7703
RO	Control	1.5325	0.0109
RPE	Control	1.14405	0.26895
iPSCs	RP13	1.18665	0.27235
KiO	RP13	0.88775	0.15125
RO	RP13	0.41465	0.20155
RPE	RP13	3.04605	0.42435

Supplementary Table 1

Reagent	Company	Catalogue number	Stock concentration	Working concentration
1-Thioglycerol	Sigma-Aldrich	M6145		450 μM
β--Mercaptoethanol	Life Technologies	31350010	5 mM	50 μM
NuPAGE™ 4-12% Bis-Tris Midi Protein Gels	Thermofisher	WG1402A		
5’ Deadenylase	New England BioLabs	M0331S		1.25 units/μl
6 Well Thincert, 0.4μm Pore diameter, translucent	Greiner	657640		
6 Well plate	TTP	92406		
Accutase	Thermo Fisher Scientific	A1110501		
Activin A	R&D systems	338-AC-010	10 mg	100 ng/ml
AdRPMI 1640	Gibco	12633		
AMpure XP	Beckman Coulter	A63880		
Antibiotic/Antimycotic	Gibco	15240-096	100x	1x
ApaL1	New England Biolabs	R0507S		
B-27 Supplement	Gibco	17504		
Blebbistatin	Sigma-Aldrich	B0560	10mM	12.5μM
Bone morphogenetic protein 4	Fisher Scientific	11330852	10 μg	1.5nM

(BMP4)				
Bovine Serum Albumin (BSA)	Sigma-Aldrich	A9418		1%
Bovine Serum Albumin (BSA) fraction V	Gibco	15260-037	7.5%	0.1%
Cell Strainer, Blue 40 µm 40 µm cell strainer	StarLab	CC8111-0042		
Chemically defined, lipid concentrate	Life Technologies	11905031		1%
CHIR99021 GSK3 Wnt signalling inhibitor	Tocris	4423	6mM	8µM
CircLigase II	Cambio	CL9021K		
CytoTune-iPS 2.0 Sendai Reprogramming Kit	Thermo Fisher Scientific	A16517		
DC™ Protein Assay Kit II	Bio-Rad Laboratories	5000112		
TURBO™ DNase	ThermoFisher	AM2238	2 U/µL	
Dimethyl Sulfoxide (DMSO)	Sigma-Aldrich	D2650	100%	10%
Dulbecco's Modified Eagle's Medium (DMEM)/F12 +L-glutamine+15 mM HEPES	Thermo Fisher Scientific	31330-038		
FastAP Thermosensit	ThermoFisher	EF0651	1 U/µL	

ive Alkaline Phosphatase FastAP alkaline phosphatase				
FITC	Sigma-Aldrich	F7250		0.4 mg/ml
Foetal Bovine Serum (FBS)	Gibco	10270		
Forskolin	Stemcell Technologies	72112	30mM	30 μM
GeneArt™ Precision gRNA Synthesis Kit	Invitrogen	A29377		
Genetic Analysis Kit	Stemcell Technologies	07550		
Genomic DNA extraction kit	Qiagen	51304		
GlutaMAX	Life Technologies	35050087		1%
Glutaraldehyde	Sigma-Aldrich	G6257		2%
GoTaq DNA polymerase	Promega	M3001		
Ham's F-12 Nutrient Mix	Thermo Fisher	21765037		45%
Heparin	Stemcell Technologies	07980	2mg/ml	1μg/ml
Human recombinant bFGF	PeprotechThermo Fisher Scientific	100-18B	250 ug	8ng/ml
IGEPAL® CA-630 (NP-40)	Sigma-Aldrich	I8896	100%	1%
ImmEdge pen	VectorLabs	H-4000		
Immobilon®-FL PVDF Membrane	Millipore	IPFL00010		

Intercept® (PBS) Blocking Buffer	LI-COR Biosciences	927-70001		
irCLIP_ddRT primers	IDT	L3-IR-phos irCLIP_ddRT _12 irCLIP_ddRT _13 irCLIP_ddRT _14 irCLIP_ddRT _15 P3 Solexa P5 Solexa		1pmol/µl
Iscove's Modified Dulbecco's Medium (IMDM)	Life Technologies	21980065		45%
Sodium dodecyl sulfate (SDS)	Sigma-Aldrich	L6026	50G	0.1%
KaryoStat™ assay	Thermo Fisher Scientific	905403		
Knockout Serum Replacement (KOSR) – used for KOs	Gibco	10828-028	100%	10%
Knockout Serum Replacement (KOSR) – used for ROs	Life Technologies	A3181502	100%	20%
KnockOut DMEM	Life Technologies	10829018	100%	
KnockOut DMEM /F12	Gibco	12660-012		
L-glutamine	Thermo Fisher Scientific	11539876	200 mM	2 mM
Lipidure	Amsbio	CM5206		0.5% w/v

Lipidure-coated 96-well U-bottomed plate	Amsbio	AMSLCP-A-U96-6		
Magnetic protein A Dynabeads	ThermoFisher	10001D		
Matrigel Growth Factor Reduced (GFR) Basement Membrane Matrix	CORNING	354230	7.9mg/ml	0.083mg/ml
Melanin Bleach Kit	Sigma-Aldrich	SHH0021-1KT		
MEM Non-Essential Amino Acids (MEM NEAA)	Fisher Scientific	31350010	50 mM	0.1 mM
mTeSR™ Plus	Stem Cell Technologies	100-0276	100%	
N2 supplement	Life Technologies	17502001	100%	1x
Nicotinamide	Sigma-Aldrich	N0636-100G	100G	10mM
Sodium deoxycholate	Sigma-Aldrich	30970	25G	0.5%
Normal donkey serum	Sigma-Aldrich	S30-100ML	100%	10%
Nucleofection solution	Lonza	V4XP-3024		
NuPAGE loading buffer	Life Technologies	NP0002	4x	1x
NuPAGE™ 3 to 8%, Tris-Acetate, 1.0–1.5 mm, Mini Protein Gels	ThermoFisher	EA0378BOX		
OCT embessing	CellPath	KMA-0100-	125ml	

matrix		00A		
Osmium tetroxide	Sigma Aldrich	201030		2%
P5Solexa/P3 Solexa primers	VWR	733-2478		
Paraformaldehyde	Santa Cruz Biotechnology	sc-281692		4%
Phosphate Buffered Saline (PBS)	Gibco	14190		1x
Polyethylene Glycol 8000 (PEG8000)	Sigma-Aldrich	1546605		20%
Penicillin-Streptomycin (Pen-Strep)	Thermo Fisher Scientific	15140130	10,000 U/mL	1%
PET 0.4 µm pore transwell ThinCerts™	Greiner Bio-One	662631		
PFHM-II protein-free hybridoma medium	Gibco	12040077	100%	5%
Platinum™ II Hot-Start PCR Master Mix	Thermo Fisher Scientific	13000012		
PluriTest™	Thermo Fisher Scientific	A38154		
Photoreceptor Outer Segments (POSs)	InVisionBioResources	98740		
Potassium ferrocyanide	Sigma-Aldrich	60279		3%
Proteinase K	MERCK	3115887001		
RecJf endonuclease	New England BioLabs	M0264S		0.75 units/µl

Recombinant human FGF9	R&D systems	273-F9	200µg/ml	200ng/ml
ReliaPre RNA Cell Minipre System	Promega	Z6012		
Retinoic Acid	Sigma-Aldrich	R2625	50 mg	0.5 µM
Reverse transcriptase (RT)	Thermo Fisher Scientific	4374966		
RNase I	ThermoFisher	EN0601		0.8 units/ml
ROCK inhibitor (Y-27632)	CHEMDEA	CD0141	20mM	10µM
Proteus Mini Clarification Spin Column Pack	Generon	GEN-MSF500		
Sodium cacodylate	Sigma-Aldrich	C0250		0.1M
STEMdiff APEL 2 medium	Stemcell Technologies	05270		
Superfrost+ microscope slide	CellPath	MBB-0102-54A		
SuperScript cDNA Synthesis Kit	Thermo Fisher Scientific	11754-050		
Superscript IV reverse transcriptase	ThermoFisher	18090010		
SYBR Green Master Mix	Thermo Fisher Scientific	A25742		
T4 Polynucleotide Kinase	New England BioLabs	M0201S		
T4 RNA ligase I	New England BioLabs	M0204S		3 units/µl

Taab 812 hard resin	TAAB laboratory Equipment	T024		
Taurine	Sigma-Aldrich	T8691		0.1 mM
Thiocarbohydrazide	Sigma-Aldrich	223220-5G		10%
Sodium chloride (NaCL)	Sigma-Aldrich	S9888	500G	100 mM
Tris hydrochloride (Tris-HCl)	Sigma-Aldrich	10812846001	500G	50 mM
Triton-X100	Sigma-Aldrich	T8787	100%	0.1-0.3%
TRIzol® Reagent	Thermo Fisher Scientific	15596-026		
TrueCut Cas9 protein	Invitrogen	A36499		100pmol
Trypan Blue	Sigma-Aldrich	T8154		
Tryple Select	Thermo Fisher Scientific	A1217701	100 mL	10x10X
TrypLE™ Express Enzyme (used for KOs)	Thermo Fisher Scientific	12604013	10x	0.75x
Trypsin-EDTA	Sigma-Aldrich	T4171	10x	1x
Uranyl acetate	Agar Scientific	AGR1000	25 ml	1%
Vectashield mounting	VectorLabs	H-1000		
Versene (EDTA 0.02%)	Lonza	15040033BE 17–771E	0.02%	
Neurosphere dissociation kit	Miltenyi Biotec	130-095-943		

Antibody target/Dye	Species isotype raised and/or dye conjugate	Company	Catalogue number/Lot number	Dilution
ZO-1	Rabbit	Invitrogen	61-7300/ YK4137360	1:100
RPGRIP1L	Rabbit	Proteintech	55160-1-AP/00054815	1:100
DRAQ5		Biostatus	DR50200/525DR05500	1:200
NPHS1	Sheep	R&D systems	AF4269/ZMU0218101	1:500
PRPF8	Rabbit	Abcam	Ab185547/gr3229172-1	1:1,000
Anti-mouse IgG	Donkey IgG-AlexaFluor647	ThermoFisher	A31571/1613070	1:2000
Anti-sheep IgG	Donkey IgG-AlexaFluor568	ThermoFisher	A21099/2249032	1:2000
CDH1/ECAD	Mouse	BD Biosciences	610181/ 931542	1:300
ARL13B	Rabbit	Proteintech	17711-1-AP/00103765	1:1,000
CEP290	Mouse	Gift from Prof Cieran Morrison		1:100
IRDye® anti-Mouse IgG	Donkey IgG-800CW	LI-COR Biosciences	926-32212/C91023-09	1:10,000
IRDye® anti-Rabbit IgG	Donkey IgG-680LT	LI-COR Biosciences	926-68023/C90821-09	1:10,000
KI-67	Mouse	DAKO	M7240/20016758	1:100
Collagen IV	Rabbit	Abcam	Ab6586/ GR3225432-2	1:100
DAPI		Thermo Fisher Scientific	D1306/2690460	1:1000
IFT88	Rabbit	Proteintech	13967-1-AP/00049531	1:200

Hoechst	Thermo Fisher	H3570	Lot 1156367	1:2000
LTL	Direct FITC conjugate	Vector Laboratories	FL-1321/ ZF0801	1:500
Ezrin	Rabbit	Proteintech	26056-1-AP	1:100
NANOG	Direct AlexaFluor647 conjugate	Cell Signaling Technology	5448s	1:50
GT335	Mouse	AdipoGen	AG-20B-0020/ A40251903	1:1,000
TRA-1-60	Direct FITC conjugate	Millipore	FCMAB115F/LV1584913	1:50
SC35	Mouse	Santa Cruz Biotechnology Inc.,	sc-53518	1:100
PRPF8	Rabbit	Home-made	N/A	1:2,000
hBrr2 (200k)	Rabbit	Home-made	N/A	1:1,000
hSnu114 (116k)	Rabbit	Home-made	N/A	1:2,000
PRPF31 (61k)	Rabbit	Home-made	N/A	1:1,000
Phospho-PRPF31	Rabbit	Home-made	N/A	1:1,000
Phospho-SF3B1	Rabbit	Home-made	N/A	1:1,000
Recoverin	Rabbit	Millipore	ab5585/13099956	1:1,000
SNCG	Mouse	Abnova	H00006623-M01A/ L3151-2C3	1:500
RetP1	Mouse	Millipore	MAB5356/126K4753	1:200
OpsinSW	Rabbit	Millipore	ab5407/3609330	1:200
PROX1	Rabbit	Millipore	ab5475/3523252	1:1,000
AP2α	Mouse	Santa Cruz Biotechnology Inc.,	sc-12726/ E1120	1:100
CRALBP	Mouse	Abcam	ab15051/ GR3388978-2	1:100
PKCα	Rabbit	Sigma	SAB4502354/210716	1:50

Goat Anti-Rabbit IgG H&L horseradish peroxidase (HRP) conjugated secondary antibody	Rabbit	Abcam	ab205718	1:25,000
Goat Anti-Mouse IgG H&L horseradish peroxidase (HRP) conjugated secondary antibody	Mouse	Abcam	ab205719	1:25,000

Table of software:

Software and algorithms		
FastQC v0.11.4	Babraham Institute	www.bioinformatics.babraham.ac.uk/projects/fastqc
MultiQC v1.10.1	[34]	https://multiqc.info/
Trimmomatic v0.36	[35]	http://www.usadellab.org/cms/?page=trimmomatic
STAR aligner v2.7.10a	[36]	https://github.com/alexdobin/STAR
featureCounts (Subread package) v2.0.1	[37]	http://subread.sourceforge.net/
DESeq2 v1.32.0	[38]	https://bioconductor.org/packages/release/bioc/html/DESeq2.html
EdgeR v3.34.1	[39]	https://bioconductor.org/packages/release/bioc/html/edgeR.html
EnhancedVolcano v1.10.0	[40]	https://bioconductor.org/packages/release/bioc/html/EnhancedVolcano.html
rMATs turbo v4.1.2	[41]	https://github.com/Xinglab/rmats-turbo

DEXSeq v1.38.0	[42]	https://bioconductor.org/packages/release/bioc/html/DEXSeq.html
clusterProfiler v4.1.0	[24]	https://bioconductor.org/packages/release/bioc/html/clusterProfiler.html
CellRanger V 3.0.1	10x genomics	
Seurat V4.3	[43]	https://satijalab.org/seurat/

Supplementary References

1. Towns, K. V. *et al.* Prognosis for splicing factor PRPF8 retinitis pigmentosa, novel mutations and correlation between human and yeast phenotypes. *Hum Mutat* (2010) **31**, 31:E1361-76.
2. Buskin, A. *et al.* Human iPSC-derived RPE and retinal organoids reveal impaired alternative splicing of genes involved in pre-mRNA splicing in PRPF31 autosomal dominant retinitis pigmentosa. *Nat Commun* (2018) **9**:4234.
3. Regent, F. *et al.* Automation of human pluripotent stem cell differentiation toward retinal pigment epithelial cells for large-scale productions. *Sci Rep* (2019) **9**, 1–11.
4. Takasato, M. *et al.* Kidney organoids from human iPS cells contain multiple lineages and model human nephrogenesis. *Nature* (2015) **536**:238.
5. Belevich, I., Joensuu, M., Kumar, D., Vihinen, H. & Jokitalo, E. Microscopy Image Browser: A Platform for Segmentation and Analysis of Multidimensional Datasets. *PLoS Biol* (2016) **14**, e1002340.
6. Deerinck, T. J. *et al.* High-performance serial block-face SEM of nonconductive biological samples enabled by focal gas injection-based charge compensation. *J Microsc* (2018) **270**, 142–149.
7. de Sena Brandine, G. & Smith, A. D. Falco: high-speed FastQC emulation for quality control of sequencing data. *F1000 Research* (2019) **8**:1874.
8. Ewels, P., Magnusson, M., Lundin, S. & Käller, M. MultiQC: summarize analysis results for multiple tools and samples in a single report. *Bioinformatics* (2016) **32**, 3047–3048.
9. Bolger, A. M., Lohse, M. & Usadel, B. Genome analysis Trimmomatic: a flexible trimmer for Illumina sequence data. *Bioinformatics* (2014) **30**, 2114–2120.
10. Dobin, A. *et al.* Sequence analysis STAR: ultrafast universal RNA-seq aligner. *Bioinformatics* (2013) **29**, 15–21.
11. Liao, Y., Smyth, G. K. & Shi, W. Sequence analysis featureCounts: an efficient general purpose program for assigning sequence reads to genomic features. *Bioinformatics* (2014) **30**, 923–930.
12. Dyer, N. P., Shahrezaei, V. & Hebenstreit, D. LiBiNorm: an htseq-count analogue with improved normalisation of Smart-seq2 data and library preparation diagnostics. *PeerJ* (2019) **7**:e6222.
13. Love, M. I., Huber, W. & Anders, S. Moderated estimation of fold change and dispersion for RNA-seq data with DESeq2. *Genome Biol* (2014)**15**, 1–21.
14. Robinson, M. D., McCarthy, D. J. & Smyth, G. K. edgeR: a Bioconductor package for differential expression analysis of digital gene expression data. *Bioinformatics* (2010) **26**, 139–140.
15. Shen, S. *et al.* rMATS: Robust and flexible detection of differential alternative splicing from replicate RNA-Seq data. *PNAS* (2014) **111**(51):E5593-601.

16. Li, Y., Rao, X., Mattox, W. W., Amos, C. I. & Liu, B. RNA-Seq Analysis of Differential Splice Junction Usage and Intron Retentions by DEXSeq. *PLoS One* (2015) **10**:e0136653.
17. Vaquero-Garcia, J. *et al.* A new view of transcriptome complexity and regulation through the lens of local splicing variations. *Elife* (2016) **5**:e11752.
18. Trincado, J. L. *et al.* SUPPA2: Fast, accurate, and uncertainty-aware differential splicing analysis across multiple conditions. *Genome Biol* (2018) **19**, 1–11.
19. Green, C. J., Gazzara, M. R. & Barash, Y. MAJIQ-SPEL: web-tool to interrogate classical and complex splicing variations from RNA-Seq data. *Bioinformatics* (2018) **34**(2):300-302.
20. Virtanen, P. *et al.* SciPy 1.0: fundamental algorithms for scientific computing in Python. *Nat Methods* (2020) **17**(3):261-272.
21. Fang, Z., Liu, X. & Peltz, G. GSEApy: a comprehensive package for performing gene set enrichment analysis in Python. *Bioinformatics* (2023) **39**(1):btac757.
22. Ashburner, M. *et al.* Gene Ontology: tool for the unification of biology. *Nature Genetics* (2000) **25**, 25–29.
23. Jassal, B. *et al.* The reactome pathway knowledgebase. *Nucleic Acids Res* (2020) **44**(D1):D481-7.
24. Wu, T. *et al.* clusterProfiler 4.0: A universal enrichment tool for interpreting omics data. *Innovation (Camb).* (2021) **2**(3):100141.
25. Mirdita, M. *et al.* ColabFold: making protein folding accessible to all. *Nat Methods* (2022) **19**: 679–682.
26. Jumper, J. *et al.* Highly accurate protein structure prediction with AlphaFold. *Nature* (2021) **596**, 583.
27. Dlakić, M. & Mushegian, A. Prp8, the pivotal protein of the spliceosomal catalytic center, evolved from a retroelement-encoded reverse transcriptase. *RNA* (2011) **17**, 799–808.
28. Tyanova, S. *et al.* The Perseus computational platform for comprehensive analysis of (prote)omics data. *Nature Methods* (2016)**13**:731–740.
29. Georgiou, M. *et al.* Activation of autophagy reverses progressive and deleterious protein aggregation in PRPF31 patient-induced pluripotent stem cell-derived retinal pigment epithelium cells. *Clin Transl Med* (2022) **12**, e759.
30. Yildirim, A. *et al.* SANS (USH1G) regulates pre-mRNA splicing by mediating the intra-nuclear transfer of tri-snRNP complexes. *Nucleic Acids Res* (2021) **49**, 5845.
31. Smith, T., Heger, A. & Sudbery, I. UMI-tools: modeling sequencing errors in Unique Molecular Identifiers to improve quantification accuracy. *Genome Res* (2017) **27**(3):491-499.
32. The RNAcentral Consortium. RNAcentral: a hub of information for non-coding RNA sequences The RNAcentral Consortium 1-38. *Nucleic Acids Res* (2019) **47**:D221-229.
33. Yeo, G. & Burge, C. B. Maximum Entropy Modeling of Short Sequence Motifs with Applications to RNA Splicing Signals. J Comput Biol (2004) **11**(2-3):377-94.
34. Ewels, P., Magnusson, M., Lundin, S. & Käller, M. MultiQC: summarize analysis results for multiple tools and samples in a single report. Bioinformatics 32, 3047–3048 (2016).
35. Bolger, A. M., Lohse, M. & Usadel, B. Genome analysis Trimmomatic: a flexible trimmer for Illumina sequence data. Bioinformatics 30, 2114–2120 (2014).
36. Dobin, A. et al. Sequence analysis STAR: ultrafast universal RNA-seq aligner. Bioinformatics 29, 15–21 (2013).
37. Liao, Y., Smyth, G. K. & Shi, W. Sequence analysis featureCounts: an efficient general purpose program for assigning sequence reads to genomic features. Bioinformatics 30, 923–930 (2014).
38. Love, M. I., Huber, W. & Anders, S. Moderated estimation of fold change and dispersion for RNA-seq data with DESeq2. Genome Biol 15, 1–21 (2014).
39. Robinson, M. D., McCarthy, D. J. & Smyth, G. K. edgeR: a Bioconductor package for differential expression analysis of digital gene expression data. Bioinformatics 26, 139–140 (2010).

40. Blighe, K., Rana, S. & Lewis, M. EnhancedVolcano: Publication-ready volcano plots with enhanced colouring and labeling. Preprint at https://bioconductor.org/packages/devel/bioc/vignettes/EnhancedVolcano/inst/doc/EnhancedVolcano.html (2023).
41. Shen, S. et al. rMATS: Robust and flexible detection of differential alternative splicing from replicate RNA-Seq data. Proc Natl Acad Sci USA 111, E5593-601 (2014).
42. Li, Y., Rao, X., Mattox, W. W., Amos, C. I. & Liu, B. RNA-Seq Analysis of Differential Splice Junction Usage and Intron Retentions by DEXSeq. PLoS One 10, e0136653 (2015).
43. Hao, Y. et al. Integrated analysis of multimodal single-cell data. Cell 184, 3573–3587 (2021).
