# Supplementary material for: PRPF8-mediated dysregulation of hBrr2 helicase disrupts human spliceosome kinetics and 5´-splice-site selection causing tissue-specific defects
Source: Nat Commun. 2024 Apr 11;15:3138. doi: 10.1038/s41467-024-47253-0 (PMC11009313; doi:10.1038/s41467-024-47253-0)
Supplement: Supplementary file 3 — Description of Additional Supplementary Files [file 41467_2024_47253_MOESM3_ESM.pdf]

Description of Additional Supplementary Files

File Name: Supplementary Data 1
Description: Summary of clinical data of RP13 patients involved in this study. The sequences of gRNA, ssODN CRISPR/Cas9 used for the in-situ gene correction and off-target sequences, and the primers used for amplifying wild type and mutant PRPF8 sequences are also included.

File Name: Supplementary Data 2
Description: Single-cell RNA-Seq data delineating the expression of highly and differentially expressed genes for each cell cluster in RP13 and RP13-Cas9 ROs. Genes that are differentially expressed between RP13 and RP13-Cas9 rod and cone photoreceptors are listed on separate sheets. A Wilcoxon Rank Sum, two-sided test test was used, the adjusted p values (corrected for multiple comparisons) are shown in the p_val_adj column).

File Name: Supplementary Data 3
Description: Differential gene expression between RP13 and RP13-Cas9 isogenic controls as determined using DESeq2. The summary sheet shows the number of aligned reads and the percentage of each RNA biotype within the dataset. Four tissue-specific sheets show differential gene expression in iPSCs, KiOs, RPE cells, and ROs. A Wilcoxon Rank Sum, two-sided test test was used, the adjusted p values (corrected for multiple comparisons) are shown in the p_val_adj column).

File Name: Supplementary Data 4
Description: Summary of GO terms for biological processes, cellular components, and molecular functions in differentially expressed genes from iPSCs, KiOs, RPE cells, and ROs from all RP13 and RP13-Cas9 control tissues. One-sided Fisher's Exact Test with p-value adjustment for multiple comparisons (Benjamini & Hochberg) was carried out.

File Name: Supplementary Data 5
Description: Differential exon usage between RP13 and RP13-Cas9 isogenic controls. The summary sheets show the number of significantly different splicing events as determined by rMATs (padj<0.05 and inclusion difference >5%). Alternative splice events (ASEs) are either skipped exon (SE), mutually exclusive exon (MXE), alternative 5’SS (A5SS), alternative 3’SS (A3SS), or retained intron (RI). The number of significant ASEs in RPE (497 genes) and ROs (1500 genes), were greater than those in KiOs (372 genes) and iPSCs (386 genes). Four tissue-specific sheets show differential exon usage (p<0.05 and inclusion level difference > 0.05) in iPSCs, KiOs, RPE cells, and ROs. One-sided likelihood-ratio test with p-value adjustment for multiple comparisons was carried out.

File Name: Supplementary Data 6
Description: Summary of GO terms for biological processes, cellular components, and molecular functions in genes with differential exon usage from iPSCs, KiOs, RPE cells, and ROs from all RP13 and RP13-Cas9 control tissues. One-sided Fisher's Exact Test with p-value adjustment for multiple comparisons (Benjamini & Hochberg) was carried out.

File Name: Supplementary Data 7
Description: Cryptic splice event (CSE) site usage between RP13 and RP13-Cas9 isogenic controls. A greater number of significant CSEs were determined by rMATs (padj<0.05 and inclusion difference >5%) in RPE (205) and RO (106), compared to iPSCs (37) and KiO (57). Four tissue-specific sheets show differential exon usage (p<0.05 and absolute log2 fold change > 1) in iPSCs, KiOs, RPE cells, and ROs.

Enrichment analyses of these gene lists are on separate sheets and include GO terms for biological processes, cellular components, and molecular functions as well as reactome terms. Fisher's exact test was used.

File Name: Supplementary Data 8
Description: Transcripts that exhibit differential binding in PRPF8 iCLIP results between RP13 and RP13-Cas9, as determined using DESeq2. Four tissue-specific sheets show differential gene expression in iPSCs, RPE cells, KiOs and ROs.

File Name: Supplementary Data 9
Description: Enrichment analysis of differentially bound transcripts identified using PRPF8 iCLIP. Transcripts were categorised according to maxent5 score into weak, intermediate, and strong splice sites prior to enrichment analysis. Four tissue-specific sheets show differential gene expression in iPSCs, RPE cells, KiOs and ROs. Individual differentially bound transcripts in ROs were clustered in specific multiprotein complexes such as RNA polymerase II (POLR1B, POLR1E, POLR2A, POLR2B, and POLR2K), IFT-B (IFT27, IFT88, DYNC1H1, and DYNC2H1) and the BBSome (BBS1, BBS4, and ARL6). In RPE, individual transcripts were for rRNA processing (WDR3, WDR36, WDR43, NOP14), IFT-B (DYNC2H1, TTC26, and KIF3B) and the BBSome (IQCB1, BBS5, CCT2, CCT4). individual differentially bound transcripts encoded ciliary proteins for both iPSCs (IFT57, IFT88, CEP89) and KiOs (WDR35, CLUAP1, DYNC2H1).

File Name: Supplementary Data 10
Description: Differences in protein expression between RP13 and RP13-Cas9 derived ROs, RPE, and KiOs, as determined using TMT-labelling and mass spectrometry. Proteins (represented by 2 or more unique peptides) are listed with differential expression (DE) between RP13 and RP13-Cas9 control proteomes of ROs (4535 unique proteins, DE=203), RPE (4574 unique proteins, DE=56) and KiOs (4294 unique proteins, DE=4). GO and KEGG enrichment analyses of these protein lists are presented in separate sheets. Two additional sheets compare RO and RPE protein expression changes with changes in transcript expression. Two-sided t-test was used for statistical analysis.
