# Supplementary material for: PRPF8-mediated dysregulation of hBrr2 helicase disrupts human spliceosome kinetics and 5´-splice-site selection causing tissue-specific defects
Source: Nat Commun. 2024 Apr 11;15:3138. doi: 10.1038/s41467-024-47253-0 (PMC11009313; doi:10.1038/s41467-024-47253-0)
Supplement: Supplementary file 14 — Reporting Summary [file 41467_2024_47253_MOESM14_ESM.pdf]

nature portfolio

Corresponding author(s): Majlinda Lako

Last updated by author(s): 01.03.2024

Reporting Summary

Nature Portfolio wishes to improve the reproducibility of the work that we publish. This form provides structure for consistency and transparency in reporting. For further information on Nature Portfolio policies, see our Editorial Policies and the Editorial Policy Checklist.

Please do not complete any field with "not applicable" or n/a. Refer to the help text for what text to use if an item is not relevant to your study.
For final submission: please carefully check your responses for accuracy; you will not be able to make changes later.

Statistics

For all statistical analyses, confirm that the following items are present in the figure legend, table legend, main text, or Methods section.

n/a	Confirmed	
☐	☒	The exact sample size (*n*) for each experimental group/condition, given as a discrete number and unit of measurement
☐	☒	A statement on whether measurements were taken from distinct samples or whether the same sample was measured repeatedly
☐	☒	The statistical test(s) used AND whether they are one- or two-sided<br>*Only common tests should be described solely by name; describe more complex techniques in the Methods section.*
☒	☐	A description of all covariates tested
☐	☒	A description of any assumptions or corrections, such as tests of normality and adjustment for multiple comparisons
☐	☒	A full description of the statistical parameters including central tendency (e.g. means) or other basic estimates (e.g. regression coefficient) AND variation (e.g. standard deviation) or associated estimates of uncertainty (e.g. confidence intervals)
☐	☒	For null hypothesis testing, the test statistic (e.g. F, t, r) with confidence intervals, effect sizes, degrees of freedom and P value noted<br>*Give P values as exact values whenever suitable.*
☒	☐	For Bayesian analysis, information on the choice of priors and Markov chain Monte Carlo settings
☒	☐	For hierarchical and complex designs, identification of the appropriate level for tests and full reporting of outcomes
☐	☒	Estimates of effect sizes (e.g. Cohen's d, Pearson's r), indicating how they were calculated

Our web collection on statistics for biologists contains articles on many of the points above.

Software and code

Policy information about availability of computer code

Data collection: No software used

Data analysis:
FastQC v0.11.4 www.bioinformatics.babraham.ac.uk/projects/fastqc
MultiQC v1.10.1 https://multiqc.info/
Trimmomatic v0.36 http://www.usadellab.org/cms/?page=trimmomatic
STAR aligner v2.7.10 a https://github.com/alexdobin/STAR
featureCounts v2.0.1 (Subread package) http://subread.sourceforge.net/
D ESeq 2 v1.32.0 https :/ /bi oco n du ctor .org/pa ckages/ release/bi oc/htm 1/D ESeq2. htm I
Edge R v3.34.1 https ://b ioco n du ctor. org/ packages/release/bi oc/htm I/ edge R. htm I
En ha ncedVo lea no v1.10.0 https :/ /bi ocon d ucto r. o rg/ packages/rel ease/b ioc/htm I/En ha n cedVol ca no. htm I
rMATs turbo v4.1.2 https://github.com/Xinglab/rmats-turbo
D EXSeq v1.38.0 https ://bi oco nd u ctor. org/pa ckage sf re lea se/bioc/htm 1/D EXSe q. htm I
cl u sterProfi le r v 4.1.0 https :/ /bi ocon d uctor. org/packages/ re lea se/bioc/htm I/ cl usterP rofi le r. htm CellRanger V 3.0.1 IOx genomics
Seurat V4.3 https://satijalab.org/seurat/

For manuscripts utilizing custom algorithms or software that are central to the research but not yet described in published literature, software must be made available to editors and reviewers. We strongly encourage code deposition in a community repository (e.g. GitHub). See the Nature Portfolio guidelines for submitting code & software for further information.

Data

Policy information about availability of data

All manuscripts must include a data availability statement. This statement should provide the following information, where applicable:

- Accession codes, unique identifiers, or web links for publicly available datasets
- A description of any restrictions on data availability
- For clinical datasets or third party data, please ensure that the statement adheres to our policy

The trimmed FASTQ data for bulk RNA-Seq of all samples included in this study were uploaded to SRA under the accession number BioProject ID PRJNA989762 [https://www.ncbi.nlm.nih.gov/bioproject/?term=PRJNA989762] and GEO under the accession number GSE236702 [https://www.ncbi.nlm.nih.gov/geo/query/acc.cgi?acc=GSE236702]. The mass spectrometry proteomics data have been deposited to the ProteomeXchange under the accession number PXD043645 [http://www.ebi.ac.uk/pride/archive/projects/PXD043645]. The single cell RNA-Seq data have been deposited to the GEO under the accession number GSE235866 [https://0-www-ncbi-nlm-nih-gov.brum.beds.ac.uk/geo/query/acc.cgi?acc=GSE235866]. The iCLIP-Seq data have been uploaded to Annotare under the accession number E-MTAB-13171 [E-MTAB-13171 < ArrayExpress < BioStudies < EMBL-EBI].

Research involving human participants, their data, or biological material

Policy information about studies with human participants or human data. See also policy information about sex, gender (identity/presentation), and sexual orientation and race, ethnicity and racism.

Reporting on sex and gender	iPSCs used in this study were derived from three males and one female. There was no biological reason for investigating the role of sex on disease.
Reporting on race, ethnicity, or other socially relevant groupings	No such categorization was used in our analysis.
Population characteristics	Three male (aged 80, 57 and 18 years old) and one female (aged 36 years old) RP13 patients donated their dermal skin biopsy for derivation of fibroblasts which were subsequently reprogrammed to iPSCs and differentiated to all cell lineages described in this study.
Recruitment	Patients were recruited based on a family history of clinically-diagnosed retinitis pigmentosa and identification of a known pathogenic PRPF8 mutation. All participants, including unaffected or pre-symptomatic family members, gave informed consent to research studies. There was no self-selection or sex bias in ascertaining these individuals.
Ethics oversight	Informed consent for research studies, according to the protocols approved by Yorkshire and the Humber Research Ethics Committe (REC ref: 15/YH/0365), was obtained for all study participants.

Note that full information on the approval of the study protocol must also be provided in the manuscript.

Field-specific reporting

Please select the one below that is the best fit for your research. If you are not sure, read the appropriate sections before making your selection.

[X] Life sciences   [ ] Behavioural & social sciences   [ ] Ecological, evolutionary & environmental sciences

For a reference copy of the document with all sections, see nature.com/documents/nr-reporting-summary-flat.pdf

Life sciences study design

All studies must disclose on these points even when the disclosure is negative.

Sample size	To determine the optimal number of biological replicates for experiments on PRPF8 in the current proposal, we performed statistical power calculations on data obtained from our PRPF31 study The power calculations indicated that three biological replicates (three patients iPSCs and iPSC-derived cells) and three isogeneic controls were sufficient for statistical power >0.8 associated with type 1 error rate α=0.05 and a
Data exclusions	No data was excluded. Some iPSC lines do not efficiently differentiate into certain tissues (e.g. RP13-1B into retinal organoids (RO), RP13-1C into retinal pigment epithelium (RPE), and RP 13-2 int o kidney organoids)). This placed a constraint on the number of iPSC lin es that could be use d for experiment s, particularly those requiring a large amount of biological material (e .g. sufficient material for iCLIP could only be generated from two iPSC lines for RPE and RO).
Replication	All experiment al result s were replicated using iPSCs derived from multiple individuals. For most experiments, results were replicated across iPSCs derived from 3-4 individuals. Once exception was the iCLIP experiment, where sufficient material was generated from cells derived from t wo iPSC lines (see above).
Randomization	Groups formed depending on genotype.
Blinding	Blinding is not relevant to this study. This is a descriptive study and the experiments and analysis were designed to minimise risk of subjective interpretation

Reporting for specific materials, systems and methods

We require information from authors about some types of materials, experimental systems and methods used in many studies. Here, indicate whether each material, system or method listed is relevant to your study. If you are not sure if a list item applies to your research, read the appropriate section before selecting a response.

Materials & experimental systems

n/a	Involved in the study	
☐	☒	Antibodies
☒	☐	Eukaryotic cell lines
☒	☐	Palaeontology and archaeology
☒	☐	Animals and other organisms
☒	☐	Clinical data
☒	☐	Dual use research of concern
☒	☐	Plants

Methods

n/a	Involved in the study	
☒	☐	ChIP-seq
☐	☒	Flow cytometry
☒	☐	MRI-based neuroimaging

Antibodies used

Antibody target/Dye	Species isotype raised and/or dye conjugate	Company	Catalogue number/Lot number	Dilution
ZO-1	Rabbit	Invitrogen	61-7300/ YK4137360	1:100
RPGRIP1L	Rabbit	Proteintech	55160-1-AP/00054815	1:100
DRAQ5		Biostatus	DR50200/525DR05500	1:200
NPHS1	Sheep	R&D systems	AF4269/ZMU0218101	1:500
PRPF8	Rabbit	Abcam	Ab185547/gr3229172-1	1:1,000
Anti-mouse IgG	Donkey IgG-AlexaFluor647	ThermoFisher	A31571/1613070	1:2000
Anti-sheep IgG	Donkey IgG-AlexaFluor568	ThermoFisher	A21099/2249032	1:2000
CDH1/ECAD	Mouse	BD Biosciences	610181/ 931542	1:300
ARL13B	Rabbit	Proteintech	17711-1-AP/00103765	1:1,000
CEP290	Mouse	Gift from Prof Cieran Morrison		1:100
IRDye® anti-Mouse IgG	Donkey IgG-800CW	LI-COR Biosciences	926-32212/C91023-09	1:10,000
IRDye® anti-Rabbit IgG	Donkey IgG-680LT	LI-COR Biosciences	926-68023/C90821-09	1:10,000
KI-67	Mouse	DAKO	M7240/20016758	1:100
Collagen IV	Rabbit	Abcam	Ab6586/ GR3225432-2	1:100
DAPI		Thermo Fisher Scientific	D1306/2690460	1:1000
IFT88	Rabbit	Proteintech	13967-1-AP/00049531	1:200
Hoechst	Thermo Fisher	H3570	Lot 1156367	1:2000
LTL	Direct FITC conjugate	Vector Laboratories	FL-1321/ ZF0801	1:500
Ezrin	Rabbit	Proteintech	26056-1-AP	1:100
NANOG	Direct AlexaFluor647 conjugate	Cell Signaling Technology	5448s	1:50
GT335	Mouse	AdipoGen	AG-20B-0020/ A40251903	1:1,000
TRA-1-60	Direct FITC conjugate	Millipore	FCMAB115F/LV1584913	1:50
SC35	Mouse	Santa Cruz Biotechnology Inc.,	sc-53518	1:100
PRPF8	Rabbit	Home-made	N/A	1:2,000
hBrr2 (200k)	Rabbit	Home-made	N/A	1:1,000
hSnu114 (116k)	Rabbit	Home-made	N/A	1:2,000
PRPF31 (61k)	Rabbit	Home-made	N/A	1:1,000
Phospho-PRPF31	Rabbit	Home-made	N/A	1:1,000
Phospho-SF3B1	Rabbit	Home-made	N/A	1:1,000
Recoverin	Rabbit	Millipore	ab5585/13099956	1:1,000
SNCG	Mouse	Abnova	H00006623-M01A/ L3151-2C3	1:500
RetP1	Mouse	Millipore	MAB5356/126K4753	1:200
OpsinSW	Rabbit	Millipore	ab5407/3609330	1:200
PROX1	Rabbit	Millipore	ab5475/3523252	1:1,000
AP2α	Mouse	Santa Cruz Biotechnology Inc.,	sc-12726/ E1120	1:100
CRALBP	Mouse	Abcam	ab15051/ GR3388978-2	1:100
PKCα	Rabbit	Sigma	SAB4502354/210716	1:50
Goat Anti-Rabbit IgG H&L horseradish peroxidase (HRP) conjugated secondary antibody	Rabbit	Abcam	ab205718	1:25,000
Goat Anti-Mouse IgG H&L horseradish peroxidase (HRP) conjugated secondary antibody	Mouse	Abcam	ab205719	1:25,000

Validation

The CEP290 antibody was validated by Professor Cieran Morrison

Antibody Validation and cited examples of their use from manufacturer's website and published studies:
ZO-1 used for immunofluorescence experiments: PMID: 35656116, PMID: 32265257 (17 publications in total citing this antibody in immunofluorescence experiments)
RPGRIP1L used in immunofluorescence experiments: antibody validated in PMID: 33961633, Figure S2
NPHS1 used in immunofluorescence experiments: antibody validated in control and congenital nephrotic syndrome (CNS) patients organoids, PMID: 30514835, Figure 6
PRPF8 used for Western blotting of gradient fractionated snRNPs: antibody was validated in PMID: 8670905, Figure 2, and PMID: 22871813, Figure 1
PRPF8 used for immunofluorescence experiments and analysis of PRPF8 expression by Western blotting: antibody validation provided by the manufacturer website, https://www.abcam.com/products/primary-antibodies/prpf8prp8-antibody-ab79237.html
hBrr2 (200k) used for Western blotting: antibody was validated in PMID: 8670905, Figure 2
hSnu114 (116k) used for Western blotting: antibody was validated in PMID: 9233818, Figure 2
PRPF31 (61k) used for Western blotting: antibody was validated in PMID: 11867543, Figure 2
Phospho-PRPF31 used for Western blotting: antibody was validated in PMID: 20118938, Figure 2
Phospho-SF3B1 used in immunofluorescence experiments and Western blotting of purified spliceosomes: antibody was validated in PMID: 22871813, Figures 1, 4 and 5
CDH1/ECAD used for immunofluorescence experiments: Antibody validated in PMID: 11904289, PMID: 10930470 and PMC2198867.
ARL13B used in immunofluorescence experiments: Antibody validated in PMID: 36764291, PMID: 28562594 (631 publications in total citing this antibody in immunofluorescence experiments)
KI-67 used in immunofluorescence experiments: Antibody validated in PMID: 29426936 (21 publications in total citing this antibody in immunofluorescence experiments)
Collagen IV used in immunofluorescence experiments: Antibody validated in PMID: 28317912, PMID: 28158775 (10 publications in total citing this antibody in immunofluorescence experiments).
IFT88 used in immunofluorescence experiments: Antibody validated in PMID: 22425997, PMID: 21552265 (231 publications in total citing this antibody in immunofluorescence experiments). Validation was provided by the manufacturer website: https://www.ptglab.com/products/IFT88-Antibody-13967-1-AP.htm#top
LTL used in immunofluorescence experiments: Antibody validated in PMID: 38269090, PMID: 38189094 and PMID 26493500 (Figure 2) (341 publications in total citing this antibody in immunofluorescence experiments).
Ezrin used in immunofluorescence experiments: antibody validated in PMID: 35305541 and PMID: 28938000. Antibody validation provided by the manufacturer website: https://www.ptglab.com/products/Ezrin-Antibody-26056-1-AP.htm
NANOG used in flow cytometry experiments, validation performed by omitting primary antibody and replacing this with isotype control in six publications (e.g., PMID: 30315276, PMID: 29374141, PMID: 28521042).
GT335 used for immunofluorescence experiments: PMID: 34912111 (20 publications in total citing this antibody in immunofluorescence experiments). Antibody validation provided by the manufacturer website: https://adipogen.com/ag-20b-0020-anti-polyglutamylation-modification-mab-gt335.html
TRA-1-60 used in Flow Cytometry experiments: PMID: 27484861, PMID: 28521042, PMID: 29631617 (17 publications in total citing this antibody in Flow Cytometry). Validation performed by omitting primary antibody and replacing this with isotype control.
SC35 has been used in immunofluorescence experiments: antibody was validated in PMID: 22871813, Figures 3 and 4
Recoverin used in immunofluorescence experiments: antibody validated in PMID: 35298655 and PMID: 29408885.
SNCG used in immunofluorescence experiments: antibody validated in PMID: 34738615 and PMID: 32023475.
RetP1 used in immunofluorescence experiments: antibody validated in PMID: 35298655, PMID: 30873007
OpsinSW used in immunofluorescence experiments: antibody validated in PMID: 25489227, PMID: 23226298, PMID: 20671290, PMID: 19255154, PMID: 17325188 and PMID: 16565408
PROX1 used in immunofluorescence experiments: antibody validated in 57 publications in total. Antibody validation provided by the manufacturer website: https://www.merckmillipore.com/GB/en/product/Anti-Prox-1-Antibody,MM_NF-AB5475#anchor_REF
AP2α used in immunofluorescence experiments: antibody validated in PMID: 3728745495, PMID: 35935488. More than 50 publications cited this antibody in immunofluorescence experiments. Antibody validation provided by the manufacturer website: https://www.scbt.com/p/ap-2alpha-antibody-3b5
CRALBP used for immunofluorescence experiments: antibody validated in PMID:28922560, PMID: 27506453, PMID: 22892561.
PKCα used for immunofluorescence experiments: antibody validated in PMID: 28074469

Flow Cytometry

Plots

Confirm that:

- [X] The axis labels state the marker and fluorochrome used (e.g. CD4-FITC).
- [X] The axis scales are clearly visible. Include numbers along axes only for bottom left plot of group (a 'group' is an analysis of identical markers).
- [X] All plots are contour plots with outliers or pseudocolor plots.
- [X] A numerical value for number of cells or percentage (with statistics) is provided.

Methodology

Sample preparation

All in format ion on sample preparation is presented in the methods section.

RPE cells were washed with PBS and were detached from the wells using 200µl of TryplE Select (lOX) for 15 minutes. Then, control and RP 13-RPE cells were resuspended in flow buffer (PBS with 2% FBS) and were transferred into Eppendorf tubes followed by centrifugation at 300 x g. To distinguish cells from debris and unbound POSs, RPE cells were incubated with 5 mM DRAQS for 5 minutes. To quench fluorescence from unbound POSs, cells were incubated with 0.2% Trypan Blue solution for 10 minutes. RPE cells were wash ed 3 times with PBS, and cell pellets were resuspended in flow buffer.

iPSCs were dissociated by incubation with Accutase for 5 minutes at 37C. A total of lx106 single cells were resuspended in PBS supplemented with 5% fetal calf serum (FCS) and stained with anti-TRA -1 -60 antibody (direct ly conjugated to FITC, 1:50) and anti -NANOG antibody (directly conjugated to AlexFlu or647 conjugate, 1:50) for two hours at 2-8°C followed by PBS washing.

Instrument

RPE samples were run on a LSRII flow cytometer (BD Biosciences) and iPSC samples were run FACSCanto flow cytometer (BD Biosciences).

Software

RPE samples were analysed using FCS Express 7 and iPSC samples were analysed using BD FACSDiva.

Cell population abundance

At least 10,000 cells were analyzed from each sample.

Gating strategy

Initial gating of debris was performed using FSC -A vs SSC-A. In RPE ana lysis, singlets were identified using FSC-A vs FSC-H. Then fluorescence was measured using the excitation and filter indicated.

- [X] Tick this box to confirm that a figure exemplifying the gating strategy is provided in the Supplementary Information.
